# Analysis of sequential hair segments reflects changes in the metabolome across the trimesters of pregnancy

**DOI:** 10.1038/s41598-017-18317-7

**Published:** 2018-01-08

**Authors:** Thibaut D. J. Delplancke, Jamie V. de Seymour, Chao Tong, Karolina Sulek, Yinyin Xia, Hua Zhang, Ting-Li Han, Philip N. Baker

**Affiliations:** 1 Department of Obstetrics and Gynaecology, The First Affiliated Hospital of Chongqing Medical University, Chongqing, China; 2 International Joint Laboratory of Maternal and Fetal Medicine, Chongqing Medical University, Chongqing, China; 3 Liggins Institute, University of Auckland, Auckland, New Zealand; 4 The Novo Nordisk Foundation Center for Basic Metabolic Research, Faculty of Health and Medical Sciences, University of Copenhagen, Blegdamsvej, 3b, 6.6.24, Copenhagen, Denmark; 5 Department of Occupational and Environmental Hygiene, School of Public Health and Management, Chongqing Medical University, Chongqing, China; 6 College of Medicine, Biological Sciences and Psychology, University of Leicester, Leicester, United Kingdom

## Abstract

The hair metabolome has been recognized as a valuable source of information in pregnancy research, as it provides stable metabolite information that could assist with studying biomarkers or metabolic mechanisms of pregnancy and its complications. We tested the hypothesis that hair segments could be used to reflect a metabolite profile containing information from both endogenous and exogenous compounds accumulated during the nine months of pregnancy. Segments of hair samples corresponding to the trimesters were collected from 175 pregnant women in New Zealand. The hair samples were analysed using gas chromatography-mass spectrometry and liquid chromatography-mass spectrometry. In healthy pregnancies, 56 hair metabolites were significantly different between the first and second trimesters, while 62 metabolites were different between the first and third trimesters (p < 0.05). Additionally, three metabolites in the second trimester hair samples were significantly different between healthy controls and women who delivered small-for-gestational-age infants (p < 0.05), and ten metabolites in third trimester hair were significantly different between healthy controls and women with gestational diabetes mellitus (p < 0.01). The findings from this pilot study provide improved insight into the changes of the hair metabolome during pregnancy, as well as highlight the potential of the maternal hair metabolome to differentiate pregnancy complications from healthy pregnancies.

## Introduction

Pregnancy complications such as preeclampsia (PE), gestational hypertension (GH), preterm birth (PTB), fetal growth restriction resulting in a small-for-gestational-age (SGA) infant, and gestational diabetes mellitus (GDM) have adverse effects on the health of both mother and fetus during pregnancy, and thereafter. These complications are the leading causes of maternal and fetal morbidity and mortality^1,2^. Long-term effects of these complications include increased risks of cardiovascular disease, stroke, and diabetes in both mother and the developing infant^3–7^. Although substantial work has been done to investigate pregnancy complications, new insights are still needed to better understand the etiology, to improve current approaches to detection and treatment, and thus benefit the health of both mother and infant.

Metabolomics, the study of the downstream products (metabolites) from interactions on the genomic or proteomic level, is the newest addition to the “omics” research field^8–10^. Metabolomics is gaining popularity as a tool to investigate pregnancy complications, to identify metabolic biomarkers, and to gain a better understanding of the underlying mechanisms at the metabolic level. Insights obtained from metabolomic studies can be used to diagnose or screen for the pregnancy complications and so implement preventive actions or personalized treatment plans^5,11^. Due to the dynamic nature of conventional samples (urine and plasma) used in metabolomics studies, the analysis can be influenced by diverse factors such as dietary variation and daily activities. Conventional biological samples convey limitations for use in the study of long-term effects of environmental exposure on pregnancy outcomes.

Human hair grows at approximately 1 cm per month^12^ and both endogenous compounds and environmental chemicals are assimilated into hair during growth. As a result, a hair sample can provide a metabolite profile that reflects exposure over months. Two studies have previously exploited human hair as a stable specimen for identifying robust biomarkers of pregnancy disorders. Through use of gas chromatography-mass spectrometry-based (GC-MS) metabolite profiling of maternal hair samples from a cohort of 83 Singaporean women, statistically significant variations in a range of metabolites were found between cases and controls, including amino acids, amino acid derivatives, fatty acids, cofactors, and antioxidants^13^. From the results of that study, a biomarker model was produced that was capable of discriminating pregnancies complicated by fetal growth restriction (FGR) from healthy pregnancies, using five metabolites (lactate, levulinate, 2-methyloctadecanate, tyrosine, and margarate: area under the receiver operating characteristic (ROC) curve of 0.998 (95% CI: 0.992–1.000)). More recently, a similar metabolomic approach was applied for an investigation into pregnancy biomarkers of GDM^14^; hair metabolite profiles from 47 cases diagnosed with GDM were compared with 47 controls with no pregnancy complication. The hair metabolome analysis identified a significantly elevated level of adipic acid in women that developed GDM that was hypothesized to be associated with the oxidative stress environment observed in diabetes^15^.

Since hair is a stable specimen which can provide long-term metabolic information, we hypothesised that the metabolomic analysis of hair segments could be used to study the longitudinal metabolite profile across the three trimesters. In addition, we investigated whether the hair metabolome could differentiate pregnancies complicated by GDM or with an SGA infant, from healthy pregnancies.

## Results

### Clinical characteristics of participants

Clinical characteristics of the Auckland study participants are shown in Table 1. A comparison between the demographic variables of controls and complicated pregnancies (SGA and GDM) showed that placental weight, birth weight, head circumference, baby length, and maternal BMI were significantly lower in the SGA group when compared to healthy pregnancies. The first four variables are associated with SGA etiology, but maternal BMI is not directly associated with SGA, so this variable was used as a confounding factor.

**Table 1:** Clinical characteristics of the participants of healthy pregnancies, and GDM and SGA complicated pregnancies.

Characteristics		**Control (n** = **73)**	**GDM (n** = **11)**		**SGA (n** = **20)**	
				p-value		p-value
Maternal age (years)^a^		33.0 ± 5.6	32.7 ± 7.1	0.90	30.8 ± 4.5	0.06
Maternal BMI (kg/m^2^, at booking)^b^		26.2 ± 5.7	27.4 ± 5.6	0.19	23.0 ± 6.1	0.002
Gestational age (weeks)^a^		39.9 ± 1.1	38.6 ± 0.8	<0.001	39.1 ± 0.8	0.001
Placenta weight (grams)^a^		693 ± 138	676 ± 106	0.37	547 ± 106	<0.001
Birthweight (grams)^a^		3537 ± 457	3403 ± 345	0.26	2752 ± 252	<0.001
Head circumference (cm)^b^		35.3 ± 2.8	35.1 ± 1.1	0.36	33.2 ± 1.4	<0.001
Baby length (cm)^b^		51.5 ± 3.5	51.0 ± 1.7	0.13	49.0 ± 1.9	<0.001
Ethicity n (%)^c^	Asian	6 (8.2)	3 (27.3)		4 (20.0)	
	European	46 (63.0)	6 (54.5)		12 (60.0)	
	Indian	8 (11.0)	0 (0)		3 (15.0)	
	Pacific Islander	13 (17.8)	2 (18.2)	0.21	1 (5.0)	0.67
Infant Sex n (%)^c^	Male	38 (52.1)	5 (45.5)		11 (55.0)	
	Female	35 (47.9)	6 (54.5)	0.93	9 (45.0)	0.64

^a^P-value was determined by student’s t-test: ^b^P-value was determined by Mann-Whitney test;: ^c^P-value was determined by Chi-squared test. P-values represent the difference in the clinical characteristics between controls and GDM/SGA.

### The metabolite profiles, predicted metabolic pathways, and metabolic network of hair segments from healthy pregnant women between trimesters

Using the in-house MCF library and NIST14 libraries, 198 metabolites were identified and retained from the GC-MS data after the dataset clean-up procedure (See Supplementary Table 1). Using the in-house exposome library and the on-line databases of HMDB, Drug Bank, and Lipid Map, 782 metabolites were identified and retained from the LC-MS data after clean-up procedure (See Supplementary Table 2).

**Figure 1:**
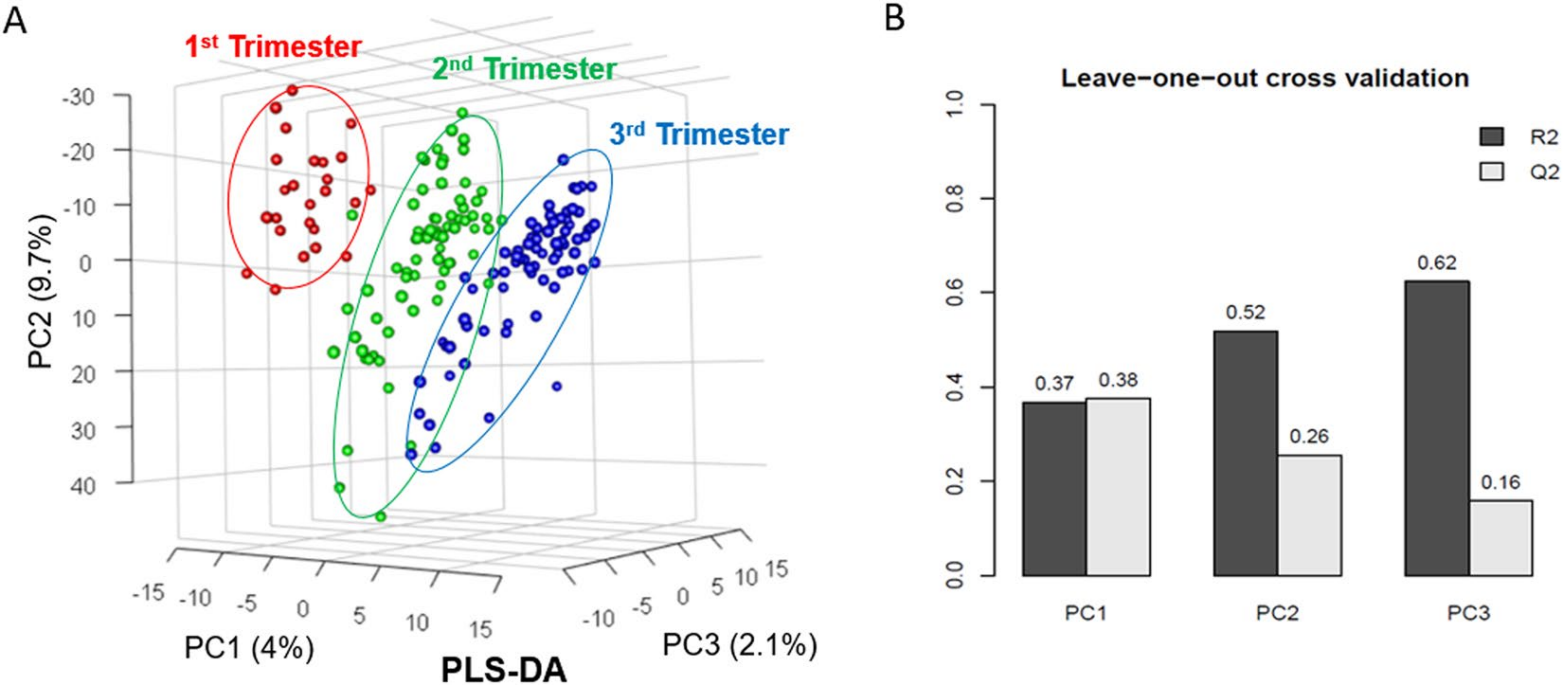
(**A**) Partial least squares-discriminant analysis (PLS-DA) of healthy pregnancy controls; first-trimester hair segments (red), second trimester (green), third trimester (blue). (**B**) Leave-one-out cross validation for PLS-DA.

A partial least squares-discriminant analysis (PLS-DA) of the hair metabolome in healthy pregnancies showed a separation (mainly by principal component 1 (PC1)) between all the trimesters, with minor overlaps (Fig. 1a). The R2 and Q2 validations of the PLS-DA model we0.64 and 0.16 for two accumulated principal components, respectively (Fig. 1b).

**Figure 2:**
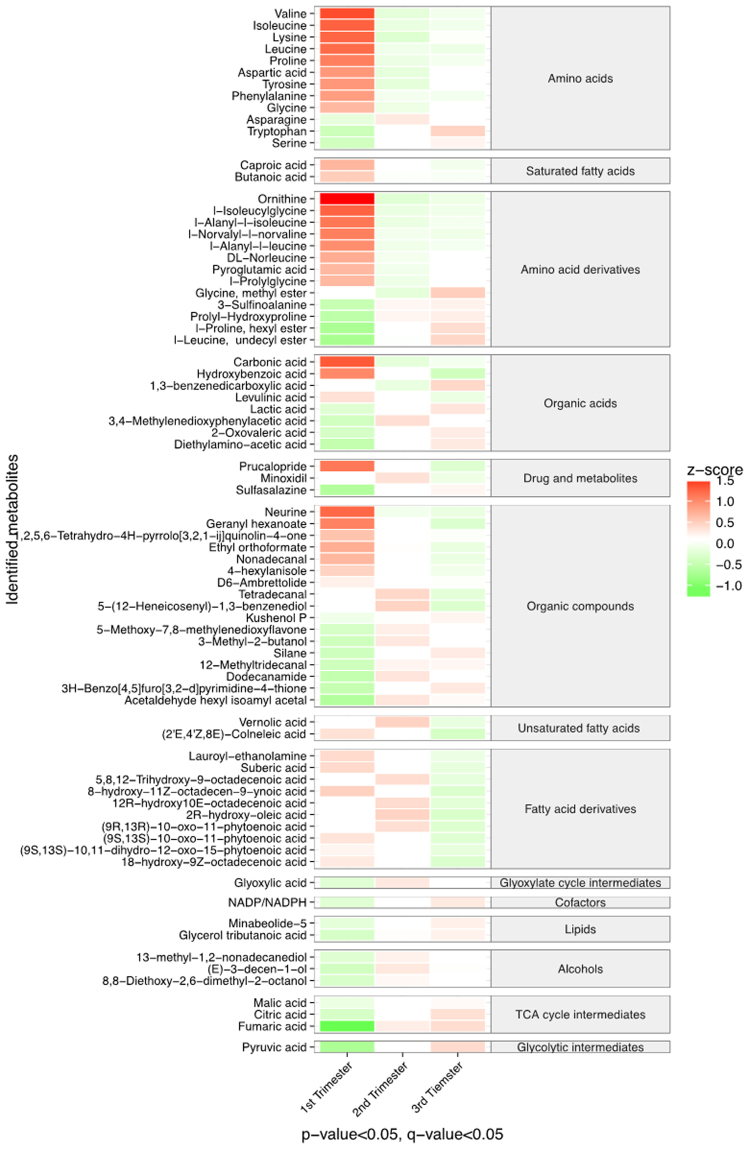
Heat map shows metabolites that differed significantly across the trimesters of normal pregnancies. Red colors represent higher metabolite concentrations, while green colors indicate lower metabolite levels. The relative concentration of metabolite was scaled to have the mean of 0 and standard deviation of 1 (z-score). Only metabolites with p-value and q-value less than 0.05 between trimesters are shown.

Among the 980 metabolites identified from the GC-MS and LC-MS analysis, a total of 78 metabolites were found to be significantly altered either when transitioning from first to second trimester or second to third trimester (p < 0.05, q < 0.05; Fig. 2). This demonstrates that the metabolic profile of hair changes substantially when transitioning across trimester of healthy pregnancy. The majority of significantly different metabolites between trimesters were amino acids and derivatives, alcohols, organic acids, organic compounds, fatty acids (saturated, unsaturated, and derivatives), drug metabolites, and tricarboxylic acid (TCA) cycle intermediates. When compared to first trimester, the amino acids and their derivatives, and approximately half of the fatty acids and organic compounds, were found in lower levels in the second and third trimesters. The other half of the fatty acids (unsaturated and derivatives) were increased when transitioning from first to second trimester and subsequently decreased when transitioning to third trimester. The TCA cycle intermediates and alcohols were elevated when transitioning from first to second and third trimesters.

**Figure 3:**
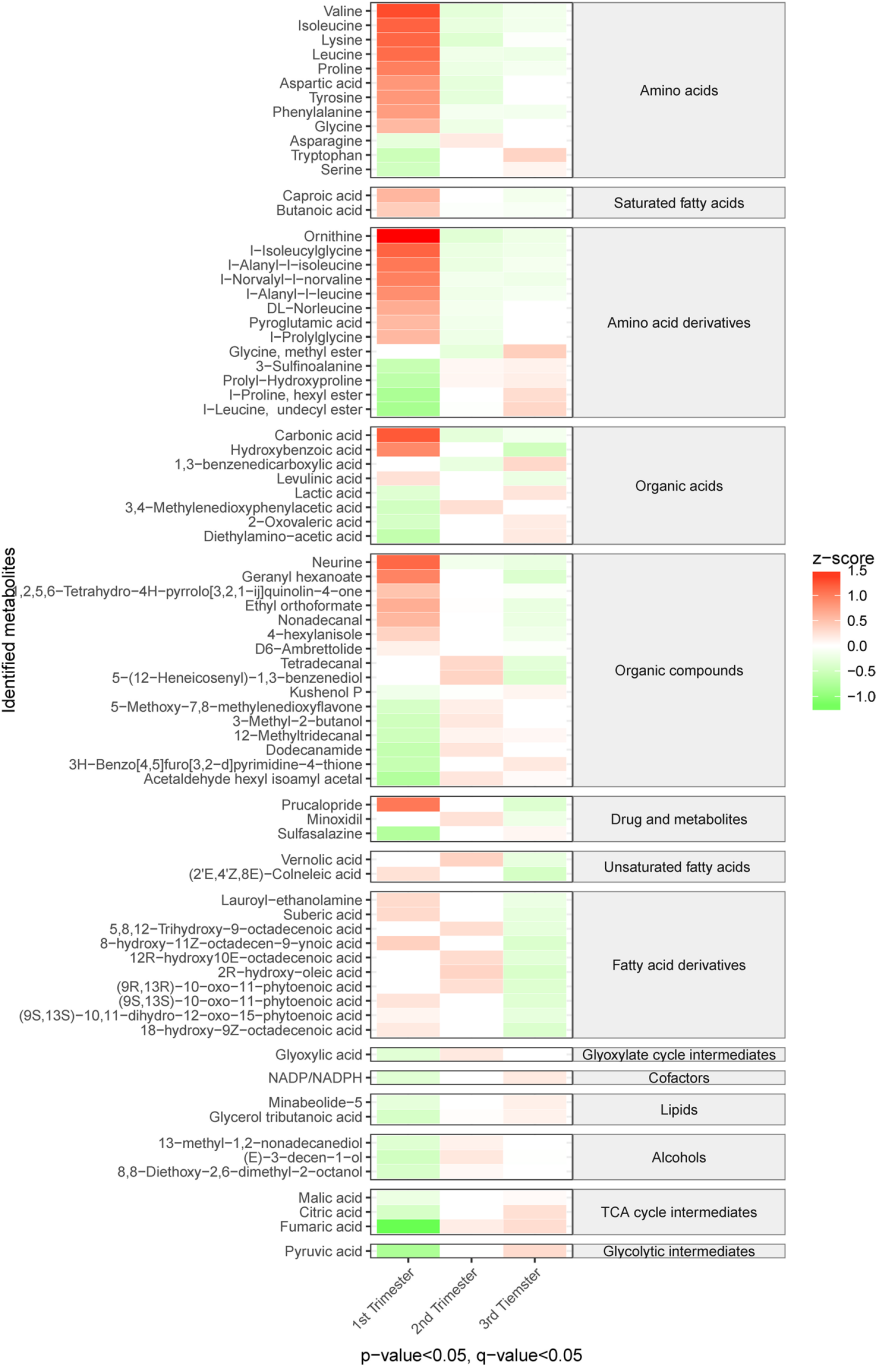
Heat map shows the altered metabolic pathways across the trimesters of normal pregnancies. Red colors represent higher metabolic activities, while green colors indicate lower metabolic activities. The metabolic activity of metabolic pathway was scaled to have the mean of 0 and standard deviation of 1 (z-score). Only metabolic pathways with p-value and q-value less than 0.05 between trimesters are shown.

The identified metabolites were analyzed with metabolic pathway analysis using the KEGG database, to search for metabolic pathways which were affected throughout pregnancy. Among the identified metabolic pathways, 44 were found to be significantly altered between the first and second trimester, and 48 were altered between the first and third trimester (p < 0.05, q < 0.05; Fig. 3). The most significantly altered metabolic pathways were related to carbohydrate metabolism, energy metabolism, xenobiotics metabolism, and amino acid metabolism. The majority of carbohydrate, energy, and xenobiotics pathways, and half of the amino acid metabolism pathways showed primarily increased activity in the second and third trimesters, when compared to the first trimester. The remaining metabolic pathways showed a lower level of activity as the pregnancy progressed.

**Figure 4:**
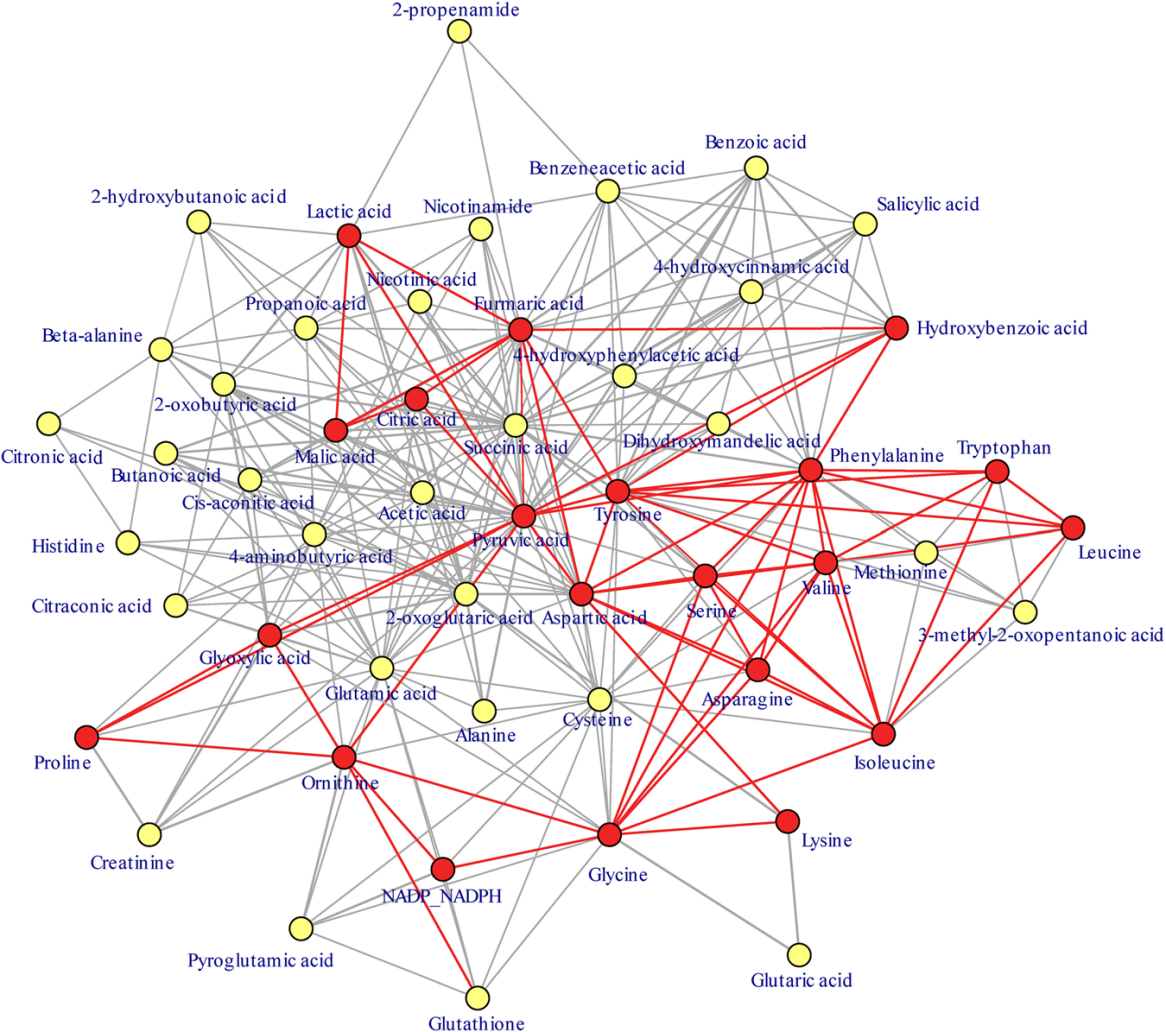
A metabolic network showing how trimester-related metabolic pathways are interconnected with common metabolites. The metabolic pathways are connected by Kamada-Kawai layout that relates the layout of metabolites to a dynamic spring system and minimizes metabolic reactions between metabolites within a metabolic network. The metabolites placed closer together will have stronger springs strength which are calculated by the inverse proportion to the square of the shortest graphical distance between two metabolites (the spring attraction was set to 68). The significantly altered metabolites throughout pregnancy are shown by the red circles and the non-significant metabolites by yellow circles. The red lines connect the significant metabolites. The topology of network is organized to have the most connected metabolites situated in the center of network.

Many significant metabolites in the metabolic network were linked with each other through diverse metabolic pathways. In the center of the metabolic network (Fig. 4), glycolytic intermediates and TCA cycle intermediates such as pyruvic acid, fumaric acid, citric acid, and malic acid were the most interconnected metabolites, connecting various metabolic pathways. The changes observed in the metabolic pathways and connections in the metabolic network indicate that energy and carbohydrate metabolism is significantly altered during pregnancy. Interestingly, aromatic amino acids such as tyrosine and phenylalanine, as well as branched-chain amino acids including isoleucine, leucine, and valine were all significantly lowered as pregnancy progressed.

Interestingly, four different types of phthalates were detected in the hair, despite vigorous water and methanol washing of the hair samples prior to processing, and adjustment for plasticizers introduced in the experiment using negative controls. Although they were not found in this study to be associated with GDM or SGA, future studies should consider exploring their origin and potential impact on maternal and offspring outcome.

### Comparison of metabolite profiles between healthy pregnancies and complicated pregnancies

**Figure 5:**
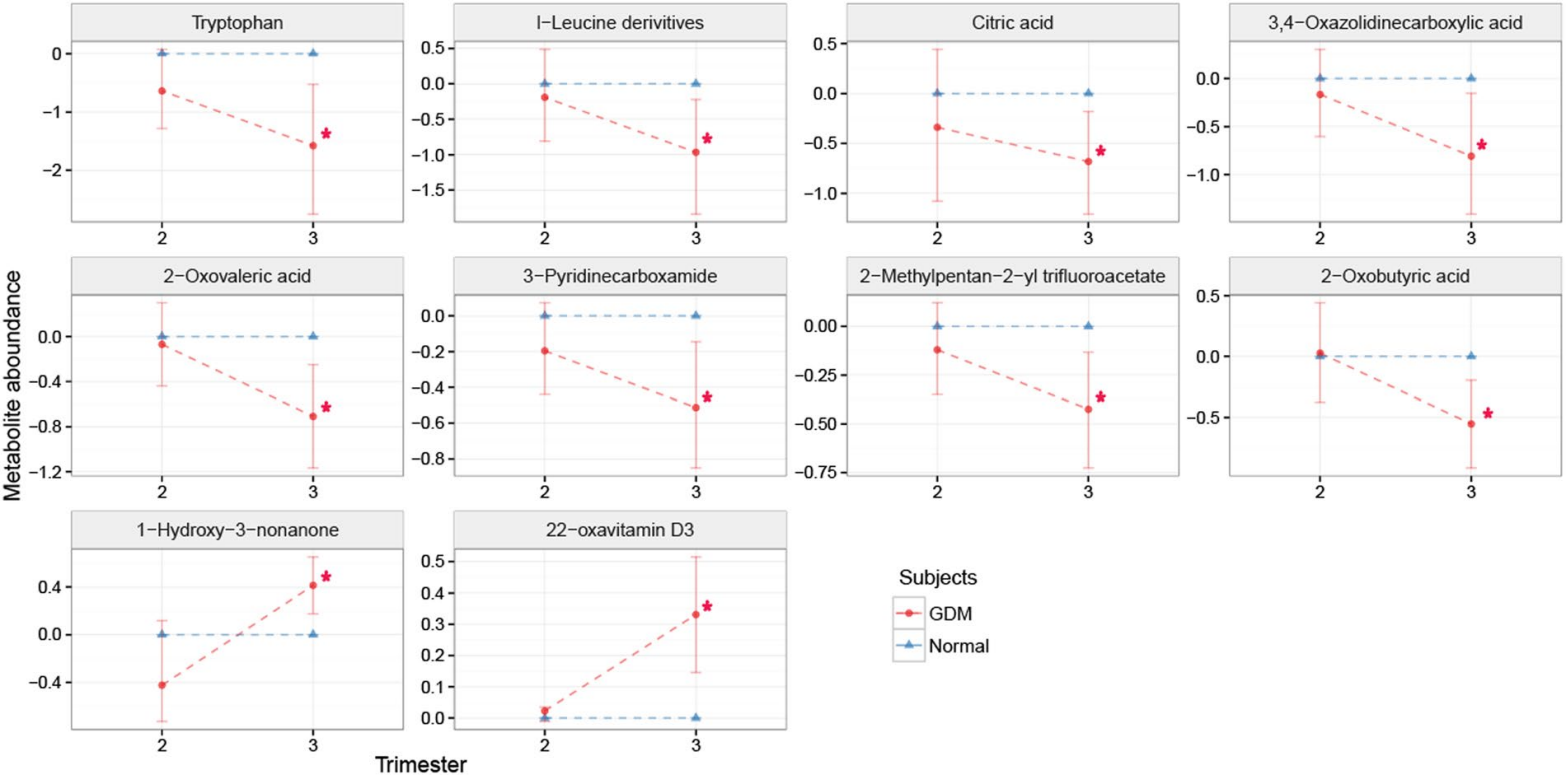
The ratio of hair metabolite levels collected from normal pregnancies as opposed to gestational diabetes mellitus (GDM) at second trimester and third trimester. Red circles represent metabolite level in maternal hairs collected from GDM. Blue triangles represent metabolite level in maternal hairs collected from normal pregnancies that were set to 0. The metabolite levels in GDM relative to the normal pregnancy were adjusted using log 2 scale. Standard deviations are shown by vertical lines. Red asterisks (*) indicate metabolites with p-values and q-values less than 0.01 and 0.17, respectively.

Due to insufficient hair length collected from mothers with pregnancy complications, only second trimester and third trimester hair segments were studied in relation to pregnancy complications. Ten metabolites were found to be significantly different between controls and GDM cases in the third trimester (Fig. 5). Metabolites that were significantly lower in the third trimester maternal hair of women who developed GDM were tryptophan, leucine, citric acid, 3,4-Oxaolidinercarboxylic acid, 2-oxovaleric acid, 3-pyridinecarboxamide, 2-methylpentan-2-yl trifluoraoacetate, and 2-oxobutyric acid, while 1-hydroxy-3–3nonanone and 22-oxavitamine D3 were found in significantly higher levels in women with GDM, when compared to controls (p-value < 0.01, q-value < 0.17).

**Figure 6:**
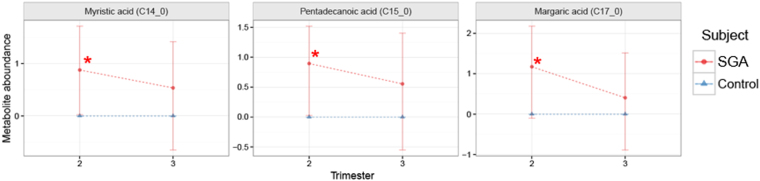
The ratio of hair metabolite levels collected from normal pregnancy as opposed to small for gestational age (SGA) at second trimester and third trimester. Red circles represent metabolite level in maternal hairs collected from SGA. Blue triangles represent metabolite level in maternal hairs collected from normal pregnancy that were set to 0. The metabolite levels relative to the normal pregnancy were adjusted using log_2_ scale. Standard deviations are shown by vertical lines. Red asterisks (*) indicate metabolites with p-values and q-values less than 0.05 and 0.6, respectively.

Three metabolites were found to be significantly increased in the second trimester hair segments of women that developed SGA, when compared to controls (Fig. 6), all of which were saturated fatty acids (margaric, pentadecanoic, and myristic; p-value < 0.05, q-value < 0.6).

## Discussion

To the best of our knowledge, this is the first study to longitudinally investigate the maternal hair metabolome in healthy pregnancies. The primary focus in hair metabolome investigations thus far has been the study of metabolic biomarkers which can be used in prediction or screening for pregnancy complications, such as FGR^13^ and GDM^14^. Recent investigation of the maternal metabolome in healthy pregnancies (plasma and amniotic fluid) has provided valuable insights to better understand the changes in the maternal metabolome^16–19^. It is essential to understand how the maternal hair metabolome will be affected by maternal metabolism throughout healthy pregnancy to better contextualise the effects of pregnancy complications on the hair metabolome.

### Changes in the hair metabolome of healthy pregancies through the trimesters

Early pregnancy is accompanied by a fall in fasting glucose values and plasma amino acids levels; and a rise in plasma free fatty acids^20^. During the transition to late pregnancy, hepatic insulin sensitivity decreases; this is a crucial prerequisite to the altered carbohydrate, lipid, and amino acid metabolism that is essential for normal fetal development^21^.

Amino acid levels in pregnancy have previously been investigated in various tissue samples, with a majority of studies showing a decrease in maternal circulation and an increase in fetal circulation^16,17,21–24^. In this study, the majority of the amino acids decreased as pregnancy progressed (Fig. 2). There are various explanations for this observation; one likely explanation is that there is an increased placental uptake from the maternal circulation to the fetus, for glucose formation and protein synthesis required to sustain the fetal energy demands and fetal growth^20^. Orczyk-Pawilowicz *et al.* ^17^ reasoned that amino acids are crucial to regulate the energy for both mother and fetus during pregnancy and as well to maintain the TCA cycle. If there was a shortage of amino acids during pregnancy, it could influence fetal protein biosynthesis, which in turn could affect fetal growth^17^. Lindsay *et al*. (2015) observed a general decrease in branched-chain amino acids (BCAAs; leucine, isoleucine, and valine), which supports our findings of a significant reduction in the three BCAAs as pregnancy progressed^16^. An increase of tryptophan and tryptophan metabolism was observed in our study, which could be explained by increased release of albumin-bound tryptophan to the free tryptophan form in maternal circulation, to meet the increased demand for protein synthesis and fetal development^25,26^. Badawy *et al.* ^25^ observed a decrease in total tryptophan plasma levels. The same authors suggested that tryptophan is important for various mechanisms during pregnancy including protein synthesis, 5-hydroxytryptophan (5-HT) synthesis for the signaling pathways, kyurenic acid production for neuronal protection, and NAD^+^ synthesis^25^.

The energy demands of the mother and fetus increase throughout pregnancy; this may explain the observed increase in TCA cycle intermediates, and the energy and carbohydrate metabolism pathways, of which the TCA cycle is an essential part^27^. Likewise, Lindsay *et al*. (2015) observed an increase in TCA cycle intermediates in plasma throughout pregnancy^16^. In our study, beside the TCA cycle intermediates, an increased level of pyruvate was observed; this is the main substrate used to produce acetyl-CoA fueling the TCA cycle^16,17^. Indeed, our pathway analysis has shown that carbohydrate metabolism increased throughout the second and third trimester, compared to the first trimester (Fig. 3). This finding implies that pregnant woman have increased rates of gluconeogenesis to meet the increased energy demands as pregnancy progresses^17,28^.

A range of fatty acids were observed at high levels in the first and second trimester hair segments when compared to the third trimester hair segments, possibly due to the maternal system being in an anabolic state where fat depots are amassed via lipogenesis. When the late-pregnancy period is presented, lipolysis of maternal fat depots increases to meet the increased energy demands of the mother and the increased fetal growth rate^20,28–30^. An increase in fatty acid and energy demands of the fetus through placental uptake may explain the decreased levels of fatty acids and increased lipid metabolism found in our study during third trimester, and previously observed in past studies^29,30^.

### Hair metabolome of pregnancy complications

In the second trimester hair segments of women who delivered SGA babies, three unsaturated fatty acids were significantly increased when compared to women whose pregnancies were uncomplicated. Two of the three fatty acids (myristic acid and margaric acid) identified in this study were also found to be higher in the hair of women that were diagnosed with FGR in the previous hair metabolomics study by Sulek *et al.* ^13^. The placental supply of fatty acids from maternal circulation to the fetus is essential for fetal growth. High levels of maternal circulating fatty acids might imply an impaired placental function in regards to fatty acid transfer to the fetus^29,30^. Horgan *et al*. (2011) also observed decreased fetal and increased maternal plasma fatty acid levels in SGA, in accordance with our results^3^. It is also possible that increased levels of fatty acids in the maternal metabolome might suggest an inadequate source of other fuels from the maternal diet to meet energy demands, and hence an increased need for lipolysis to produce fuels for the developing fetus^31^.

We observed lower levels of the amino acids tryptophan and leucine in the second and third trimester hair segments of women who developed GDM. The leucine findings in this study are particularly interesting, as previous observational research has demonstrated a link between increased levels of plasma branched chain amino acids and diabetes (BCAA; leucine, isoleucine, and valine), in contrast with our findings^7,32,33^. However, intervention studies have shown a beneficial effect of leucine supplementation for reducing obesity and improving glycaemic control in mice^33,34^. It is evident that further research needs to be conducted to understand the complex relationship between leucine and GDM.

Two of the organic acids (2-oxovaleric acid and 2-oxobutyric acid) found to be significantly lower in hair segments of women who developed GDM are also involved in energy metabolism and the degradation of amino acids; if there were low levels of amino acids in the women with GDM then there is likely to be less substrate available for degradation products to be formed^35,36^.

A previous study investigating the urine metabolome of Chinese women found that tryptophan metabolism was significantly higher in women who developed GDM, as evidenced by an increased number of metabolites associated with tryptophan degradation^37^. Lower levels of tryptophan in the women who developed GDM in our study support Law *et al*. (2017)’s findings^37^.

### Limitations

The sample size of the groups with complicated pregnancies was low in this pilot study and as such, future studies should be performed to validate these findings in a larger cohort. In addition, no information was collected at recruitment concerning hair chemical treatments which may be a limitation to this study: A study published after our cohort had been established found that the hair metabolome can be affected by chemical treatments such as dying, bleaching, and perming^38^. Future studies should consider extracting information on a participant’s past hair treatment using a questionnaire at the time of sample collection.

A limitation of the analytical methodology employed was that both the LC-MS method and sample preparation chosen favoured the detection of basic compounds; acidic compounds could only be detected if they were present in high concentrations. Ideally, this method would be combined with a liquid-liquid extraction in an acid aqueous phase. However, we had insufficient hair biomass to perform both extraction methods in the current study.

## Conclusion

The present study is the first to demonstrate that hair segmentation of the same strand of hair is capable of discriminating between trimester of pregnancy, providing an interesting insight into the transition of early pregnancy to mid/late-pregnancy. We demonstrated that the hair metabolome altered over the course of pregnancy, and can reflect changes in levels of amino acids, TCA cycle intermediates, fatty acids, cofactors, vitamin-related metabolites, and xenobiotics. The hair metabolome analysis also identified potential candidates for GDM and SGA biomarkers. The results from this pilot study require validation using large pregnancy cohorts.

## Methods

### Chemicals and reagents

Potassium hydroxide (1 M), sodium hydroxide (1 M), sodium bicarbonate (50 mM), anhydrous sodium sulphate, sulphuric acid (3 M), and 2,3,3,3-d4-alanine were purchased from Sigma-Aldrich (St. Louis, USA). Cambridge Isotope Laboratories (Andover, USA) supplied the three other internal standards; 2,2,4,4-d4-citric acid, 2,3,4,5,6-d5-phenylalanine, and 3,3-d2-tyrosine. Methanol (Optima grade) was bought from Adamas-beta® (Shanghai, China); chloroform from Chongqing Xinan Chemical Reagent Company (Chongqing, China); pyridine from Merck (Darmstadt, Germany); and methyl chloroformate (MCF) from Shandong Huayang Pesticide Chemical Industry Group (Shandong, China).

### Study Participants

Women were recruited at Auckland City Hospital and Green Lane Hospital in Auckland, New Zealand. The participants of this pilot study consisted of 175 pregnant women with healthy pregnancies or pregnancies complicated by PE, GH, GDM, PTB, or SGA. Demographic information was collected from the participants, including maternal BMI, date of birth, smoking and alcohol consumption, pregnancy and medical history. Ethics approval was granted by the Northern Health and Disability Ethics Committee (13/NTA/7) and all methods were performed in accordance with the relevant guidelines and regulations. Informed consent was acquired from each participant.

Hair samples of cases with uncertain complication status (n = 29) or with multiple pathologies (n = 14) were excluded in order to focus on the known pathologies and their association with the hair metabolome. Hair samples which were below the biomass lower limit of 0.5 mg (n = 15) were excluded from analysis.

Women were diagnosed with GDM through an Oral Glucose Tolerance Test (OGTT), using the International Association of the Diabetes and Pregnancy Study Groups criteria (equal or exceeded the plasma glucose thresholds for fasting ≥5.1 mmol/L; or two hours after a 75 g glucose load, ≥8.5 mmol/L)^39^. Gestational hypertension was diagnosed according to criteria determined by the International Society for the Study of Hypertension in Pregnancy (systolic blood pressure ≥140 mmHg and/or diastolic blood pressure ≥90 mmHg on at least two occasions)^40^. If proteinuria was found in addition to hypertension, then preeclampsia was diagnosed^41^. SGA was defined as birth weight below the 10th customized centile^42^, and preterm birth was defined as delivery before 37 weeks of gestation^43^. The hair samples from women who developed PE (n = 2), GH (n = 5), and PTB (n = 2) were excluded from further analyses due to the small number of cases.

### Hair sample collection and storage

Hair samples were collected from pregnant women between the 34th and 37th weeks of gestation. Hair strands were taken from the occipital area, 0.5 cm away from the scalp. The hair samples were placed in aluminum foil which was then stored in a zip-lock bag at −20 °C.

### Hair sample preparation for analysis

**Figure 7:**
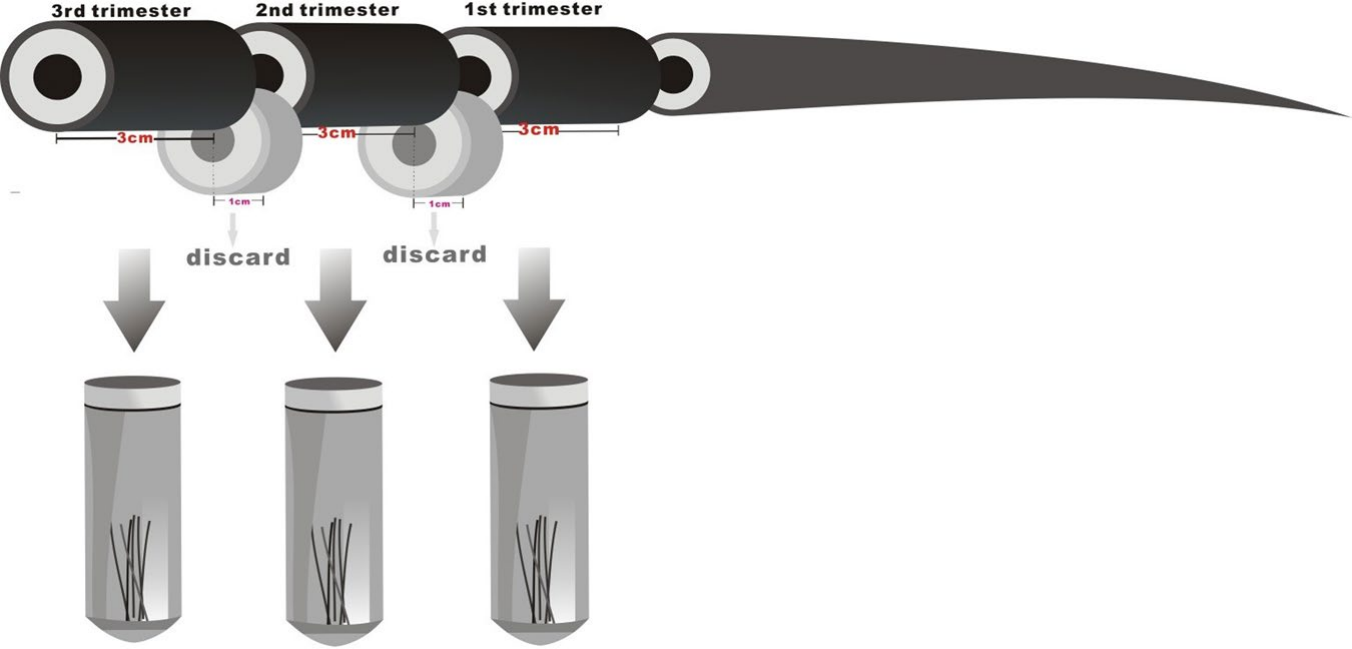
Representation of the hair segmentation.

The hair samples for the first segment (0–3 cm, third trimester) were cut and placed into a Falcon tube (Fig. 7)^44,45^. The next 1 cm segment (3–4 cm) represented combined segments of the third and second trimester which were cut and excluded. The samples for the second segment (4–7 cm, second trimester) were cut and placed into a Falcon tube. From 25 selected control samples (healthy pregnancies) a first-trimester segment (8–11 cm) was collected, after excluding a 1 cm segment. The motive for excluding the combining segments was that these could influence the results representing the different trimesters.

All hair segments underwent the sample preparation procedure described in Sulek *et al.* ^13^ and He *et al.* ^14^. Firstly, the segments were washed twice with ultrapure water and methanol. Between 0.5–5.5 mg of hair was weighed inside silanized glass vials and the weights were recorded. The samples were then incubated in 400 µL of potassium hydroxide (1 M) with 20 µL of the internal standard mix (2,3,3,3-d4-alanine (10 mM), (2,2,4,4-d4-citric acid (10 mM); 2,3,4,5,6-d5-phenylalanine (10 mM); 3,3-d2-tyrosine (2 mM)), for 16 hours at 54 °C. After alkaline hydrolysis, the extracts were neutralized by adding 67 µL of 3 M sulphuric acid and 1 mL of methanol to precipitate the salts and proteins. Centrifuging for 10 minutes at 1500 rpm resulted in the separation of the precipitate from the supernatant. 350 µL of the supernatant was then transferred into three Eppendorf tubes, for each sample. The quality control (QC) samples were prepared by pooling all the remaining supernatant (approx. 250 µL) from each sample into one tube, mixing, and then dividing into 350 µL aliquots. All the extracts were evaporated to dryness by a rotary evaporator for 6 hours and stored at −80 °C. Negative controls were prepared by subjecting empty tubes to the same process as the samples and QCs.

### Chemical derivatization

Before GC-MS analysis, the samples were chemically derivatized by the methyl chloroformate protocol published by Smart *et al.* ^46^. The dried hair extract was resuspended in 200 µL of sodium hydroxide (1 M) then transferred to a silanized tube. To the resuspended extract, a volume of 167 µL of methanol and 34 µL of pyridine were added. To initiate the derivatization, 20 µL of MCF was added and then mixed on a vortex for 30 seconds; this was then repeated. After the derivatization by MCF, 400 µL chloroform was added and mixed for 10 seconds. Subsequently, 400 µL of bicarbonate (50 mM) was added and mixed for 10 seconds. The sample was then centrifuged for 2 minutes at 2000 rpm to separate the aqueous layer from the organic layer. After the centrifugation, the aqueous layer was discarded. Anhydrous sodium sulphate was added before transferring the derivatized samples to glass inserts which were placed into GC vials for GC-MS analysis.

### Gas chromatography-mass spectrometry (GC-MS) analysis

The derivatized hair extracts were analyzed with an Agilent 7890B gas chromatograph coupled to a 5977 A mass spectrometer with electron-impact ionization operating at 70 eV. The dimensions and phase of the ZB-1701 GC capillary column employed was 30 m × 250 μm id × 0.15 μm with 5 m guard column and 14%-Cyanopropylphenyl-86%-Dimethylpolysiloxane (Phenomenex). The parameters used for hair GC-MS analysis were based on Smart *et al.* ^46^. A 1 μL amount of derivatized sample was injected into the inlet by the Agilent 7693 autosampler. The inlet temperature was set to 290 °C and operated in pulsed splitless mode at 180 kPa for 1 minute. The helium gas flow through the GC-column was set at a constant flow of 1.0 mL/min. The GC oven temperature was started at 45 °C for 2 minutes and was then increased to 180 °C at 9 °C/min, which was held for 5 minutes. Subsequently, the oven temperature was increased at 40 °C/min to 220 °C and held for 5 minutes and then it was increased again at 40 °C/min to 240 °C, for 11.5 minutes. The final increase was at 40 °C/min to 280 °C where it was held for 2 minutes. The transfer interface was kept at 250 °C, the ion source at 250 °C, and the quadrupole at 130 °C. The mass spectrometry detector was turned on after 5.5 minutes of analysis with a mass range between 30–550 amu.

### Liquid chromatography-mass spectrometry (LC-MS) analysis

Before LC-MS analysis, the samples, quality controls (QCs) and blanks went through an optimized liquid-liquid extraction based on the protocol published by Miguez-Framil *et al.* ^47^. In summary, the dried hair extracts were resuspended in 400 µl sodium hydroxide (0.5 M) and transferred to silanised tubes. Next, 600 µl of n-Hexane/ethyl acetate (7:3) mixture was added, vortexed for 1 min, centrifuged for 10 min, and then the extract was transferred to a clean silanized tube. The second extraction was performed using 600 µL of the n-Hexane/ethyl acetate (7:3) mixture, a 1 min vortex, 10 min centrifugation, and transfer of the organic extract to the same silanized tube with the previous extract. The extract was evaporated to dryness using a rotary evaporator (SpeedVac) for 30 min. The dried extract was then resuspended in 100 µL of methanol and transferred to a glass insert which was placed into vials for LC-MS analysis.

A Waters UPLC system was used to perform the liquid chromatographic (LC) separation, which was carried out at 45 °C with a Waters ACQUITY BEH-C18 column (2.1 mmx50 mm, 1.7 μm, 134 Å). Water with 0.5% formic acid (A) and acetonitrile (B) were used as mobile phases. The linear gradient was as followed: 0 to 1 min, 5 to 50% mobile phase B; 1 to 10 min, 50 to 90% B; 10 to 12 min, 90 to 99% B; 12 to 13 min, attained 99% B; at 13 min a steep decrease to 5% B until end of run at 15 min. The injection volume was 5 µl and the flow rate was 0.4 ml/min. The autosampler (Sample Manager-FTN, ACQUITY UPLC Class, Waters) was maintained at 4 °C. The mass spectrometer, which was a QTOF (XEVO G2-S), was used for analysis and operated in positive electrospray (ESI+) mode. The scan range of the mass spectrometer was 50 to 1200 m/z with a data acquisition rate of 1.0 s. The capillary voltage was set at 3.00 kV(ESI+) and the ion source temperature was set at 120 °C, cone gas flow and desolvation gas flow were 50 (L/Hr) and 800 (L/Hr), respectively.

The MS/MS data was acquired in a data-independent acquisition MS^*E*^ mode with dynamic range scanning from 50 to 1200 m/z with a data acquisition rate of 1.0 s. The collision energy was 6 V at low-energy and was ramping from 20 to 45 V at low-energy with dewell time of 0.2 s. Lock mass was performed real time to correct mass accuracy using Leucine Encephaline at a sampling frequency of 20 s. The instrumental mass calibration was implemented using sodium formate (0.5 mM) in 9:1 propanol/water prior the analysis of each batch. The mass resolution and mass accuracy, determined by Leucine Encephalin, was 30,000 FWHM and 5 ppm.

### Data extraction, normalization, enrichment analysis, and statistical analysis

The GC-MS data from the samples, QCs, and negative controls were deconvoluted by the Automated Mass Spectral Deconvolution and Identification system (AMDIS) software^48,49^ and chromatographic peaks were identified using our in-house MCF mass spectra library and National Institute of Standards and Technology (NIST14) library (See Table S1). The compound identification was confirmed by matching both mass spectrum and the corresponding chromatographic retention time against in-house MCF mass spectrometry library, but only the mass spectra were implicated for compound identification when using the NIST 14 MS library. For relative quantification and metabolite identification, xcms R-based software was used. After manual removal of false positive and false negative identifications, the data was first normalized using the four internal standards (chosen based on the highest correlation of internal standard for each metabolite in QC samples), and adjustment for daily batch effects was performed by median centering using the QC samples. The final normalization was conducted using the hair biomass.

Progenesis QI software was used to extract the LC-MS data and perform metabolite identification. The identification was accomplished using an in-house exposome database and external databases such as Drug Bank, Human Metabolome Database, and Lipid Maps database. After data extraction, metabolite identification, and removal of false positive and false negatives, the data was normalized by support vector regression using R-based software MetNormalizer, to remove intra- and inter- batch effects^50^. The final normalization was conducted using the hair biomass.

Metabolites were log-transformed before testing for significant differences between groups using multivariate analysis of analysis of variance (ANOVA), logistic regression, and pairwise comparison via Tukey’s HSD test in R. Findings were adjusted for multiple comparison testing using the Storey and Tibshirani procedure in R, and these results are reported as q-values^51^. P-value and corresponding q-value < 0.05 was considered statistically significant. The Pathway Activity Profiling R package (PAPi) using the Kyoto Encyclopedia of Genes and Genomes (KEGG) online database was applied to determine which metabolic pathways differed significantly across trimesters. PAPi estimates an activity score for each metabolic pathway found in the KEGG database based on the number of metabolites detected from each pathway and their relative concentration. As a result, the activity score represents the probability that a metabolic pathway is active and allows statistical analysis of metabolic pathway activity between groups in a metabolomics dataset. The significance for KEGG analysis was determined by pairwise comparison via Tukey HSD Test. The illustration of heatmaps and metabolic networks were generated by the ggplot2 and igraph R packages, respectively^52,53^.

The datasets generated and analysed during the current study are available from the corresponding author on reasonable request.

## Electronic supplementary material

Supplementary Material

## References

[1] Villar J (2006). Preeclampsia, gestational hypertension and intrauterine growth restriction, related or independent conditions?. American journal of obstetrics and gynecology.

[2] Cruz-Lemini M (2012). Risk of perinatal death in early-onset intrauterine growth restriction according to gestational age and cardiovascular Doppler indices: a multicenter study. Fetal diagnosis and therapy.

[3] Horgan RP (2011). Metabolic profiling uncovers a phenotypic signature of small for gestational age in early pregnancy. Journal of proteome research.

[4] Bellamy L, Casas JP, Hingorani AD, Williams DJ (2007). Pre-eclampsia and risk of cardiovascular disease and cancer in later life: systematic review and meta-analysis. Bmj..

[5] Dessì A, Marincola FC, Fanos V (2015). Metabolomics and the great obstetrical syndromes–GDM, PET, and IUGR. Best Practice & Research Clinical Obstetrics & Gynaecology.

[6] van Vliet E (2013). Metabolomics reveals metabolic alterations by intrauterine growth restriction in the fetal rabbit brain. PloS one.

[7] Huynh J, Xiong G, Bentley-Lewis R (2014). A systematic review of metabolite profiling in gestational diabetes mellitus. Diabetologia.

[8] Wishart DS (2007). Current progress in computational metabolomics. Briefings in bioinformatics.

[9] Hollywood K, Brison DR, Goodacre R (2006). Metabolomics: current technologies and future trends. Proteomics.

[10] Bouatra S (2013). The human urine metabolome. PloS one.

[11] Cecatti JG (2016). Use of metabolomics for the identification and validation of clinical biomarkers for preterm birth: Preterm SAMBA. BMC pregnancy and childbirth.

[12] Cooper GA, Kronstrand R, Kintz P (2012). Society of Hair Testing guidelines for drug testing in hair. Forensic Science International.

[13] Sulek K (2014). Hair metabolomics: identification of fetal compromise provides proof of concept for biomarker discovery. Theranostics.

[14] He X (2016). Maternal hair metabolome analysis identifies a potential marker of lipid peroxidation in gestational diabetes mellitus. Acta diabetologica.

[15] Inouye M, Mio T, Sumino K (2000). Dicarboxylic acids as markers of fatty acid peroxidation in diabetes. Atherosclerosis.

[16] Lindsay KL (2015). Longitudinal metabolomic profiling of amino acids and lipids across healthy pregnancy. PloS one.

[17] Orczyk-Pawilowicz M (2016). Metabolomics of human amniotic fluid and maternal plasma during normal pregnancy. PloS one.

[18] Luan H (2014). Pregnancy-induced metabolic phenotype variations in maternal plasma. Journal of proteome research.

[19] Diaz SO (2012). Following healthy pregnancy by nuclear magnetic resonance (NMR) metabolic profiling of human urine. Journal of proteome research.

[20] Hadden DR, McLaughlin C (2009). Normal and abnormal maternal metabolism during pregnancy. Seminars in Fetal and Neonatal Medicine..

[21] Young M, Prenton MA (1969). Maternal and fetal plasma amino acid concentrations during gestation and in retarded fetal growth. BJOG: An International Journal of Obstetrics & Gynaecology.

[22] Battaglia FC, Regnault TRH (2001). Placental transport and metabolism of amino acids. Placenta.

[23] Di Giulio AM (2004). Plasma amino acid concentrations throughout normal pregnancy and early stages of intrauterine growth restricted pregnancy. The Journal of Maternal-Fetal & Neonatal Medicine.

[24] Schoengold DM, Parlett RC (1978). Free amino acids in plasma throughout pregnancy. American journal of obstetrics and gynecology.

[25] Badawy AAB (2015). Tryptophan metabolism, disposition and utilization in pregnancy. Bioscience reports.

[26] Wang M (2016). Normal pregnancy-induced amino acid metabolic stress in a longitudinal cohort of pregnant women: novel insights generated from UPLC-QTOFMS-based urine metabolomic study. Metabolomics.

[27] Owen OE, Kalhan SC, Hanson RW (2002). The key role of anaplerosis and cataplerosis for citric acid cycle function. Journal of Biological Chemistry.

[28] Herrera E (2000). Metabolic adaptations in pregnancy and their implications for the availability of substrates to the fetus. European journal of clinical nutrition.

[29] Saleh AK, Al-Muhtaseb N, Gumaa KA, Mubarak A, Shaker MS (1989). Maternal, amniotic fluid and cord blood metabolic profile in normal pregnant and gestational diabetics during recurrent withholding of food. Hormone and metabolic research.

[30] Herrera E, Amusquivar E, Lopez-Soldado I, Ortega H (2006). Maternal lipid metabolism and placental lipid transfer. Hormone Research in Paediatrics.

[31] Park S, Park JY, Lee JH, Kim SH (2015). Plasma levels of lysine, tyrosine, and valine during pregnancy are independent risk factors of insulin resistance and gestational diabetes. Metabolic syndrome and related disorders.

[32] Suhre K (2014). Metabolic profiling in diabetes. Journal of Endocrinology.

[33] Zhang Y (2007). Increasing dietary leucine intake reduces diet-induced obesity and improves glucose and cholesterol metabolism in mice via multimechanisms. Diabetes.

[34] Guo K, Yu YH, Hou J, Zhang Y (2010). Chronic leucine supplementation improves glycemic control in etiologically distinct mouse models of obesity and diabetes mellitus. Nutrition & metabolism.

[35] Database, H. M. 2-oxovaleric acid. [cited2017 10/04/2017]; Available from: http://www.hmdb.ca/metabolites/HMDB01865.

[36] Database, H. M. *2-oxobutyric aci*d. [cited2017 10/04/2017]; Available from: http://www.hmdb.ca/metabolites/HMDB00005.

[37] Law KP, Han TL, Mao X, Zhang H (2017). Tryptophan and purine metabolites are consistently upregulated in the urinary metabolome of patients diagnosed with gestational diabetes mellitus throughout pregnancy: A longitudinal metabolomics study of Chinese pregnant women part 2. Clinica Chimica Acta.

[38] Joo KM (2016). Metabolomic analysis of amino acids and lipids in human hair altered by dyeing, perming and bleaching. Experimental dermatology.

[39] American Diabetes Association. Diagnosis and classification of diabetes mellitus. *Diabetes care*, **37**(Supplement 1), S81–S90 (2014). 10.2337/dc14-S081 24357215

[40] Brown MA, Lindheimer MD, de Swiet M, Assche AV, Moutquin JM (2001). The classification and diagnosis of the hypertensive disorders of pregnancy: statement from the International Society for the Study of Hypertension in Pregnancy. Hypertens Pregnancy.

[41] North RA (2011). Clinical risk prediction for pre-eclampsia in nulliparous women: development of model in international prospective cohort. Bmj.

[42] McCowan L, Stewart AW (2004). Term birthweight centiles for babies from New Zealand’s main ethnic groups. Australian and New Zealand Journal of Obstetrics and Gynaecology.

[43] WHO. [cited 2016 21/12/2016]; Available from: http://www.who.int/mediacentre/factsheets/fs363/en/.

[44] Gizlenti S, Ekmekci TR (2014). The changes in the hair cycle during gestation and the post‐partum period. Journal of the European Academy of Dermatology and Venereology.

[45] Harkey MR (1993). Anatomy and physiology of hair. Forensic Science International.

[46] Smart KF, Aggio RB, Van Houtte JR, Villas-Bôas SG (2010). Analytical platform for metabolome analysis of microbial cells using methyl chloroformate derivatization followed by gas chromatography-mass spectrometry. Nature protocols.

[47] Míguez-Framil M (2014). An improved method for the determination of ∆ 9-tetrahydrocannabinol, cannabinol and cannabidiol in hair by liquid chromatography–tandem mass spectrometry. Microchemical Journal.

[48] Grapp M, Maurer HH, Desel H (2016). Systematic forensic toxicological analysis by GC‐MS in serum using automated mass spectral deconvolution and identification system. Drug testing and analysis.

[49] Buszewska-Forajta M, Kordalewska M, Bartosińska E, Siluk D, Kaliszan R (2016). Compound identification in metabolomics: a study with the use of two different GC data processing systems. Journal of Analytical Chemistry.

[50] Shen X (2016). Normalization and integration of large-scale metabolomics data using support vector regression. Metabolomics.

[51] Storey JD, Tibshirani R (2003). Statistical significance for genomewide studies. Proceedings of the National Academy of Sciences.

[52] Wickham, H. *ggplot2: elegant graphics for data analysis*. (Springer, New York, 2009).

[53] Nepusz GCAT, Csárdi G (2006). The igraph software package for complex network research. Complex Systems.

