# Author Correction: Analysis of sequential hair segments reflects changes in the metabolome across the trimesters of pregnancy

**DOI:** 10.1038/s41598-020-60642-x

**Published:** 2020-02-21

**Authors:** Thibaut D. J. Delplancke, Jamie V. de Seymour, Chao Tong, Karolina Sulek, Yinyin Xia, Hua Zhang, Ting-Li Han, Philip N. Baker

**Affiliations:** 1 Department of Obstetrics and Gynaecology, The First Affiliated Hospital of Chongqing Medical University, Chongqing, China; 2 International Joint Laboratory of Maternal and Fetal Medicine, Chongqing Medical University, Chongqing, China; 3 Liggins Institute, University of Auckland, Auckland, New Zealand; 4 The Novo Nordisk Foundation Center for Basic Metabolic Research, Faculty of Health and Medical Sciences, University of Copenhagen, Blegdamsvej, 3b, 6.6.24, Copenhagen, Denmark; 5 Department of Occupational and Environmental Hygiene, School of Public Health and Management, Chongqing Medical University, Chongqing, China; 6 College of Medicine, Biological Sciences and Psychology, University of Leicester, Leicester, United Kingdom

Correction to: *Scientific Reports* 10.1038/s41598-017-18317-7, published online 08 January 2018

The HTML and PDF versions of this Article contain an incorrect version of Figure 3.

The correct Figure 3 appears below as Figure 1.

**Figure 1:**
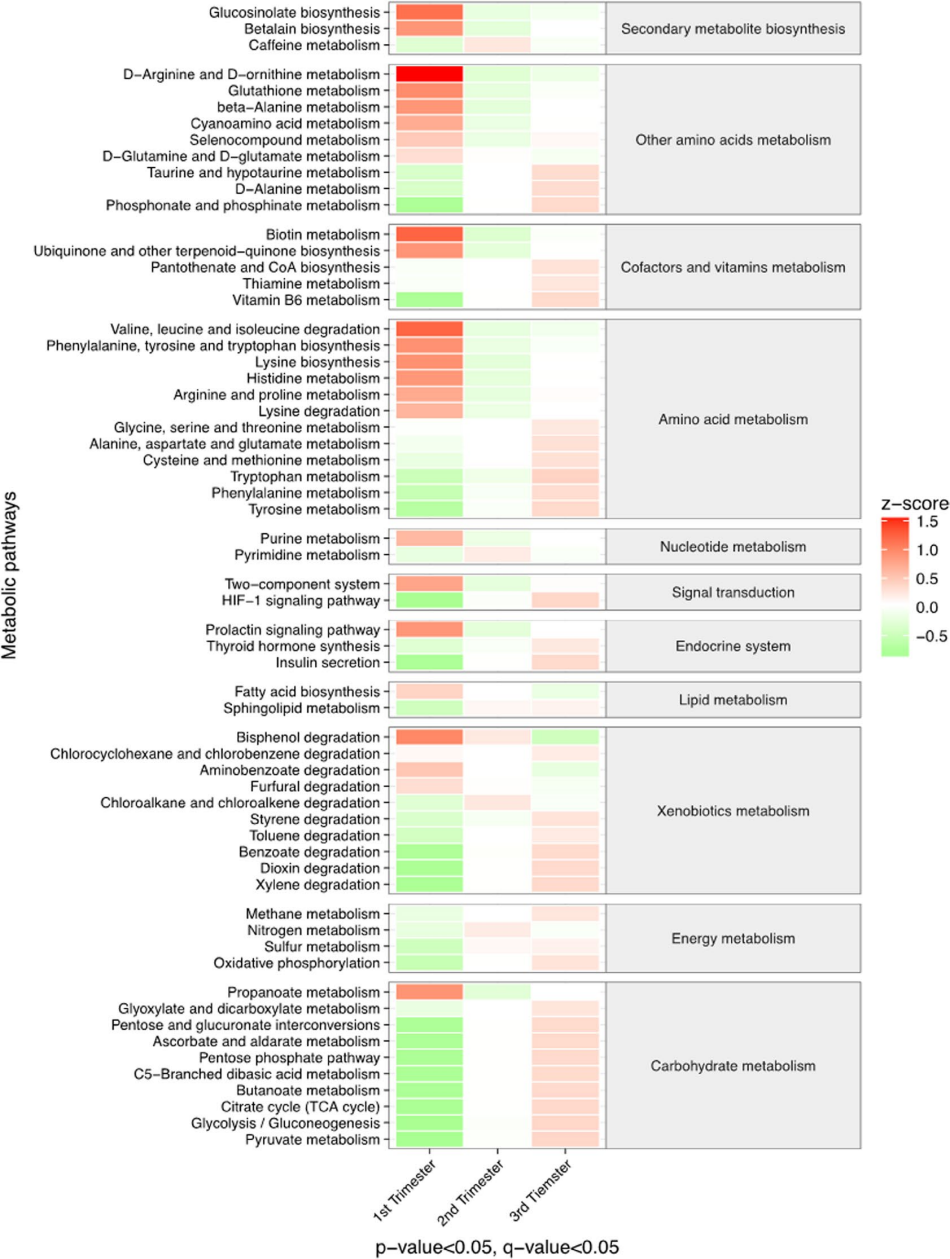
.

