# Supplementary material for: Analysis of sequential hair segments reflects changes in the metabolome across the trimesters of pregnancy
Source: Sci Rep. 2018 Jan 8;8:36. doi: 10.1038/s41598-017-18317-7 (PMC5758601; doi:10.1038/s41598-017-18317-7)
Supplement: Supplementary file 1 — Supplementary Material [file 41598_2017_18317_MOESM1_ESM.pdf]

Supplementary Material

Analysis of sequential hair segments reflects changes in the metabolome across the trimesters of pregnancy

Thibaut D.J. Delplancke[1,2+], Jamie V. de Seymour[3+], Chao Tong[1,2+], Karolina Sulek[4], Yinyin Xia[5], Hua Zhang[1,2], Ting-Li Han[1,2,3*], Philip N. Baker[1,2,6]

[1] Department of Obstetrics and Gynaecology, The First Affiliated Hospital of Chongqing Medical University, Chongqing, China;
[2] International Joint Laboratory of Maternal and Fetal Medicine, Chongqing Medical University, Chongqing, China;
[3] Liggins Institute, University of Auckland, Auckland, New Zealand;
[4] The Novo Nordisk Foundation Center for Basic Metabolic Research, Faculty of Health and Medical Sciences, University of Copenhagen, Blegdamsvej, 3b, 6.6.24, Copenhagen, Denmark;
[5] Department of Occupational and Environmental Hygiene, School of Public Health and Management, Chongqing Medical University, Chongqing, China;
[6] College of Medicine, Biological Sciences and Psychology, University of Leicester, Leicester, United Kingdom.

[+] These authors contributed equally to this work.

*Correspondence: Ting-li Han, Email: t.han@auckland.ac.nz

Supplementary Table 1. Metabolites identified by GC-MS and their classification

Classification	Compounds
**Alcohols**	4-pentene-2-ol[e]; 1-hexadecanol[d]; 1-dodecanol[c]; cyclohexanol[d]; 2-ethyl-1-hexanol[d]; n-tridecan-1-ol;
**Amino acid and derivatives**	alanine[a]; glycine[a]; valine[a]; leucine[a]; isoleucine[a]; proline[a]; threonine[a]; aspartic acid[a]; asparagine[a]; serine[a]; beta-alanine[a]; glutamic acid[a]; methionine[a]; phenylalanine[a]; cysteine[a]; ornithine[a]; lysine[a]; histidine[a]; tyrosine[a]; tryptophan[a]; 1-aminocyclopropane-1-carboxylic acid[a]; 4-aminobutyric acid (GABA)[a]; 2-aminobutyric acid[a]; pyroglutamic acid[a]; 1-aminocyclopentanecarboxylic acid[d]; N-(carboxymethyl)-L-alanine[a]; glycyl-l-leucine[c]; glycyl-l-proline[c]; l-alanyl-l-isoleucine[e]; l-alanyl-l-leucine[d]; l-isoleucylglycine[c]; l-leucyl-l-alanine[d]; l-leucylglycine[c]; l-norleucyl-l-norleucine[d]; l-norvalyl-l-norvaline[e]; l-prolylglycine[d]; N-methoxy-N-methylamino-methyl-(S)-proline[e]; N.alpha.-acetyl-l-lysine[e]; 1-alanyl-1-proline[d]; d-prolyl-d-proline[c];
**Anti-oxidants**	Glutathione;
**Co-factors**	NADP_NADPH[a];
**Drugs**	ethambutol[d];
**Branched, Saturated, and Unsaturated Fatty acids**	10,13-dimethyltetradecanoic acid[a]; caprylic acid[a]; dodecanoic acid[a]; myristic acid[a]; pentadecanoic acid[a]; margaric acid[a]; stearic acid[a]; heneicosanoic acid[a]; tridecanoic acid[d]; tetradecanoic acid[e]; butanoic acid[e]; hexanoic acid[d]; trans-vaccenic acid[a]; 9-octadecenoic acid[e]; 2-butenoic acid[e];
**Glycolytic intermediates**	pyruvic acid[a];
**Glyoxylate cycle intermediates**	glyoxylic acid[a];
**Organic acids**	2-oxobutyric acid[a]; 2-oxovaleric acid[a]; malonic acid[a]; 3-methyl-2-oxopentanoic acid[a]; 4-Methyl-2-oxopentanoic acid[a]; levulinic acid[a]; lactic acid[a]; glutaric acid[a]; benzoic acid[a]; citraconic acid[a]; 2-hydroxybutyric acid[a]; citramalic acid[a]; adipic acid[a]; dimethyl aminomalonic acid[a]; suberic acid[a]; azelaic acid[a]; salicylic acid[a]; hydroxybenzoic acid[a]; dipicolinic acid[a]; 4-hydroxyphenylacetic acid[a]; 4-hydroxycinnamic acid[a]; acetic acid[c]; 1-methylpyridin(2H)-2-one-5-carboxylic acid[e]; 1-propene-1,2,3-tricarboxylic acid[c]; 1,3-benzenedicarboxylic acid[e]; 1,4-benzenedicarboxylic acid[c]; 2-(1-hydroxy-1-methylethyl)pyrrolidine-1-carboxylic acid[e]; 2-(4-fluoro-6-oxo-1,6-dihydro-pyrimidin-2-ylamino)-propionic acid[d]; 3,4-oxazolidinecarboxylic acid[d]; 4-pyridinecarboxylic acid[d]; benzenepropanoic acid[e]; carbamic acid[d]; hexanedioic acid[c]; N-hydroxy-N-ethylcarbamic acid[e]; phthalic acid[d]; propanoic acid[e]; pyrrolidine-2-one-trans-4,5-dicarboxylic acid[e]; benzeneacetic acid[c]; but-2-enedioic acid[c]; 3-oxazolidinecarboxylic acid[e]; 2-oxomalonic acid[e]; 3,4-methylenedioxyphenylacetic acid[e]; 2-pyrrolidinecarboxylic acid[e]; carbonic acid[e];
**Organic compounds**	benzaldehyde[d]; dodecane[c]; tetradecane[d]; tridecane[a]; 4-octene[e]; 2-propenamide[e]; 3-pyridinecarboxamide[e]; 4-amino-3-ethyl-2-thioxo-2, 3-dihydro-thiazole-5-carboxylic acid methylamide[e]; 1-phenyl-1-propanamine[e]; benzenamine[e]; creatinine[a]; ethyl 2-(ethoxycarbonyloxy) ethylcarbamate[e]; 3,4-dihydroxymandelic acid[e]; 2,4-di-tert-butylphenol[a]; 4,4'-dimethoxybenzil[e]; 4-hexylanisole[e]; 2,6-difluoropyridine[e]; ethane[c]; pentafluoropropionic acid[e]; trichloroacetic acid[d]; 1,3-dioxane[e]; diethylamino-acetic acid[c]; 5-methyluracil[e]; benzene[e]; 2-methylpentan-2-yl trifluoroacetate[d]; anthracene[e]; 3H-benzo[4,5]furo[3,2-d]pyrimidine-4-thione[e]; 5-(β-bromoallyl)-5-

	(1-methylbutyl) barbituric acid[e]; 5-Hydroxy-2-methylthiopyrimidine[e]; 2H-tetrazole[e]; N-allyl morpholine[e]; 1-(2-Thienylmethyl)-2-thiourea[e]; 1H-indene[d]; 3-piperidinol[d]; 5,10-diethoxy-2,3,7,8-tetrahydro-1H,6H-dipyrrolo[1,2-a:1',2'-d]pyrazine[e]; 5,6-epoxy-6-methyl-2-heptanone [e]; ethyl N-(2-methoxyethyl)alaninate[e]; N,N-diethylglycine methyl ester[e]; pyrrolo[1,2-a]pyrazine-1,4-dione[e]; pyrrolo[1,2-a]pyrazine-3-propanamide[e]; 1-oxa-3,4-diazacyclopentadiene[e]; 5-cyano-4-methoxyamino-7-phenyl-hept-6-enoic acid[e]; 5H-imidazo(2,1-a)isoindole[e]; dimethyl ethylidenemalonate[e]; methyl-2-ethoxyacetate[e]; dibutyl phthalate[a]; dimethyl phthalate[c]; ethyl 2,5,8,11,14,17-hexaoxanonadecan-19-oate[e]; ethyl orthoformate[e]; isobutyl methyl phthalate [c]; bis(2-ethylhexyl) phthalate[c]; methyl 4-tert-butylbenzoate[c]; 4H-benzo[f]pyrrolo[1,2-a][1,4]diazepine[e];
**Tricarboxylic acid (TCA) cycle intermediates**	oxalic acid[a]; fumaric acid[a]; succinic acid[a]; malic acid[a]; 2-oxoglutaric acid[a]; cis-aconitic acid[a]; citric acid[a];
**Vitamins**	nicotinic acid[a]; nicotinamide[a].

[a]In-house MCF library identification match factor ≥75%; [b]In-house MCF library identification match factor below 75%; [c]NIST14 library identification match factor ≥85%; [d]NIST14 library identification match factor between 85 and 75%; [e]NIST14 library identification match factor below 75%. Higher match % were used for NIST14 library because it is less accurate compared to in-house MCF library.

Supplementary Table 2. Metabolites identified by LC-MS and their classification.

Classification	Compounds	Adducts	m/z
**Alcohols and derivatives**	3-Methyl-2-butanol	2M+ACN+H	218.211
	Hexan-2-ol	2M+ACN+H	246.242
	octane-1,2-diol	2M+ACN+H	334.295
	Hydroxycitronellol	M+ACN+H	216.195
	(R,Z)-Tridec-6-en-2-ol	M+ACN+H	240.231
	Tetradecanol	M+ACN+H	256.263
	5E-Tetradecen-1-ol	M+Na	235.204
	2,6,8,12-Tetramethyl-2,4-tridecadien-1-ol	M+H	253.252
	7E-Tetradecen-1-ol	M+Na	235.203
	14-methyl-1-hexadecanol	M+ACN+H	298.31
	11-methyl-1,2-heptadecanediol	M+ACN+H	328.321
	icosane-1,3-diol	M+ACN+H	356.353
	13-methyl-1,2-nonadecanediol	M+ACN+H	356.352
	Octadecanol	M+ACN+H	312.326
	4E,6Z,10Z-Hexadecatrien-1-ol	M+H-H2O, M+ACN+H	278.247
	(R)-Dihydrocitronellol	2M+ACN+H	358.368
	1-Hydroxy-3-nonanone	2M+ACN+H	358.296
	phthiocerol	M+ACN+H	526.517
**Amino acid and derivatives**	Isoleucyl-Proline	M+H-H2O	211.143
	(2S,2'S)-Pyrosaccharopine	M+H-2H2O	223.109
	Prolyl-Hydroxyproline	M+ACN+H	270.144
	L-cis-3-Amino-2-pyrrolidinecarboxylic acid	M+H-H2O	113.07
	Threoninyl-Isoleucine	M+H-2H2O	197.128
	5-Hydroxy-L-tryptophan	M+H, M+2Na-H	221.091
	gln-tyr	M+H-2H2O	274.118
	L-cis-Cyclo(aspartylphenylalanyl)	M+ACN+H	304.129
	N-methylphenylalanine	M+H	180.101
	Cysteinyl-Methionine	M+H-2H2O	217.046
	Prolyl-Tryptophan	M+ACN+Na	365.158
	Tryptophyl-Glycine	M+H-2H2O	226.097
	Lysyl-Valine	M+H-H2O	228.171
	Serinyl-Cysteine	M+2Na-H	253.023
	Indoleacetyl glutamine	M+H-H2O	286.118
	Phenylalanyl-Alanine	M+H-2H2O	201.103
	Tyrosyl-Threonine	M+H-2H2O	247.109
	Lysyl-Asparagine	M+2Na	153.063
	Gamma-glutamyl-Aspartate	M+Na	284.086

	Tryptophyl-Hydroxyproline	M+H-H2O, M+H, M+Na	300.134
	L-Tryptophan	M+H-H2O	187.086
	Leucyl-Lysine	M+H-2H2O	224.176
	Glutamyl-Arginine	M+H-H2O, M+H	285.144
	Isoleucyl-Phenylalanine	M+H-H2O	261.159
	Gamma-glutamyl-Histidine	M+ACN+H	325.161
	Aspartyl-Hydroxyproline	M+Na	269.074
	(5-L-Glutamyl)-L-amino acid	M+Na	241.079
	Tryptophyl-Glutamine	M+ACN+H	374.184
	Tryptophyl-Lysine	M+Na	355.173
	Glutaminyl-Asparagine	2M+Na	543.214
	Glycyl-Tryptophan	2M+Na	545.21
	Asparaginyl-Valine	M+2Na-H	276.093
	Alanyltryptophan	2M+Na	573.242
	Alanyl-Isoleucine	2M+K	443.226
	Glutaminyl-Proline	M+ACN+Na	307.137
	Diphthine	2M+K	638.338
**Cosmetic and metabolites**	4-amino-MX	M+Na	290.111
**Drug and metabolites**	Tegafur	M+H-2H2O	165.047
	Nepafenac	M+H	255.113
	4-Hydroxy-estazolam	M+ACN+H	352.095
	Hydroxylated N-acetyl desmethyl frovatriptan	M+H-2H2O	254.129
	Ciclopirox	M+ACN+H	249.16
	Tetracaine	M+Na	287.174
	Sulfasalazine	M+2H	200.041
	Desmethylnortriptyline	M+H-H2O	232.147
	Orciprenaline-3-O-sulfate	M+H-H2O	274.073
	Tofisopam	M+2H	192.102
	Aprobarbital	M+H-2H2O	175.086
	NPC	M+K	557.182
	5'-Hydroxymethyl meloxicam	M+ACN+Na	431.046
	Venlafaxine	M+H-H2O, M+H	278.211
	Nimesulide	M+H-H2O	291.044
	Pyridostigmine	2M+H	363.202
	(S)-Reticuline	M+K	368.127
	(S)-MHD	M+H	255.112
	Ibudilast	M+H	231.148
	Didesmethyl doxepin	M+H-2H2O	216.116
	Oxaprozin	M+ACN+H	335.139

Minoxidil	M+ACN+H	251.161
Cetirizine	M+H-H2O	371.151
Levofloxacin	M+ACN+H	403.177
Fenoprofen	M+ACN+H	284.129
Doxylamine	M+H	271.18
Cinacalcet	M+ACN+H	399.205
Pentazocine	M+2Na-H	330.181
Marimastat	M+H-2H2O	296.197
N-Desmethylcitalopram	2M+3H2O+2H	324.159
Rimantadine	2M+ACN+Na	422.352
Pseudoephedrine	2M+H	331.237
3-Hydroxymonoethylglycinexylidide	M+ACN+H	264.17
Ropivacaine	M+H-H2O	257.2
Dapiprazole	M+H	326.233
Carboxy-ibuprofen	M+H-2H2O	201.09
Bupivacaine	M+H-H2O	271.216
N-Desmethylpromazine	M+H	271.125
Levobupivacaine	M+H-H2O	271.216
Pentamidine	M+ACN+Na	404.207
Sufentanil	M+H-H2O	369.198
Vorinostat	M+H-2H2O	229.134
Levallorphan	M+ACN+H	325.227
Tapentadol	M+H	222.184
Mupirocin	M+H-H2O	483.297
o-O-glucuronide rosiglitazone	M+H-2H2O	514.13
Eletriptan N-oxide	M+H	399.173
Stanozolol	M+H-H2O	311.248
Linagliptin	M+2H	237.124
Epinastine	M+ACN+Na	313.143
Bevantolol	M+H-2H2O, M+Na	310.18
Diethylpropion	M+ACN+Na	269.163
Ethopropazine	M+H	313.173
Dydrogesterone	M+H	313.217
Nabumetone	M+H	229.122
Trisodium citrate dihydrate	M+H-H2O	276.992
Benzphetamine	M+ACN+H	281.202
Norethindrone	M+H	299.2
Diphenhydramine N-glucuronide	M+K, M+ACN+H	474.25
Gentian Violet	M+K, M+2Na-H	411.206
Practolol	M+H, M+Na	267.171

Tocainide	M+ACN+H	234.16
Rabeprazole	M+ACN+Na	423.146
Dolutegravir	M+H	420.138
5-Hydroxydantrolene	M+ACN+H	372.093
Eflornithine	2M+Na	387.162
Pargyline	2M+K	357.173
Orphenadrine	M+ACN+H	311.212
Propofol glucuronide	M+H-H2O	337.166
Valganciclovir	M+H-H2O, M+ACN+H	337.163
Prucalopride	M+H	368.172
Cyclandelate	M+H	277.18
meta-O-Dealkylated flecainide lactam	M+ACN+Na	410.131
Dienogest	M+H	312.196
Ethionamide sulphoxide	M+H-H2O	165.048
Flavoxate	M+H-H2O	374.176
Carfentanil	2M+3H2O+2H	408.237
Ethylmorphine	M+ACN+Na	377.185
nor-Levomethadyl acetate	M+ACN+Na	403.236
Risperidone	M+H-2H2O, M+H	375.198
Enoximone	2M+K	535.089
6-allyl-8b-Carboxy-ergoline	2M+K	631.268
Propafenone	M+ACN+H	383.232
2-Hydroxydesogestrel	M+K	365.188
Debrisoquine	M+H, 2M+K	389.186
Gestrinone	2M+K	655.318
Cyclizine	M+2Na-H	311.15
Pirenzepine	M+H, M+ACN+H	352.178
Ecabet	M+H-H2O	363.162
Carbamazepine iminoquinone	2M+H	415.144
Levobetaxolol	M+ACN+Na	371.232
Metoprolol	M+Na	290.173
Dronabinol	M+H	315.232
3'-Azido-3'-deoxy-5'- O-beta-D-glucopyranuronosylthymidine	M+Na	466.119
Propoxyphene	M+ACN+Na	403.236
Fimasartan	M+Na	524.219
N-Desmethyl sildenafil (UK-103,320)	M+H, M+ACN+H	461.198
Nafcillin	M+ACN+Na	478.139
Trimipramine	2M+H	589.429
Zolmitriptan	M+H-H2O	270.16

	Forasartan	M+Na	439.232
	Flurandrenolide	M+ACN+H	478.261
	Hydroxystilbamidine Isethionate	M+Na	555.119
	Alfacalcidol	M+H	401.341
	Trospium	M+ACN+H	434.258
**Fatty acids and derivatives**	Lonchocarpol B	M+H+Na	233.098
	3-Methylcrotonylglycine	M+H-2H2O	122.06
	16,16-dimethyl-Prostaglandin A2	M+K	401.207
	Dichotellate A	M+ACN+H	460.34
	3,6,9-Hexadecatriene	M+2Na-H	265.19
	2-ene-Valproic acid	M+H-2H2O	107.085
	3Z-Undecene-5,7,10-triynoic acid	M+ACN+H	214.087
	15-oxo-18Z-tetracosenoic acid	M+H-2H2O, 2M+3H2O+2H	444.345
	8E-Heptadecenedioic acid	M+ACN+H	340.247
	5,7,9,11,13-tetradecapentaenoic acid	2M+K	475.224
	2-keto palmitic acid	M+ACN+H	312.252
	Caproylcholine	M+K	241.143
	2-mercapto-octadecanoic acid	M+K	355.206
	Tridecanedioic acid	M+H-2H2O	209.153
	2-Hydroxymyristic acid	M+H-H2O	227.2
	9,12,13-TriHOME	M+H-2H2O, M+ACN+Na	295.226
	6E,12E-octadecadienoic acid	M+H-2H2O	245.225
	Trans-Hexa-dec-2-enoic acid	M+H-H2O, M+H	237.22
	9-Oxooctadecanoic acid	M+H-2H2O, M+H-H2O, M+H	281.247
	Stearidonic acid	M+H	277.215
	5,8,12-Trihydroxy-9-octadecenoic acid	M+H-2H2O	295.226
	3-Hydroxyisovaleric acid	2M+ACN+Na	300.141
	Stearolic acid	M+H-2H2O, M+H-H2O, M+H	263.236
	11-Oxooctadecanoic acid	M+Na	321.239
	6R-hydroxy-tetradecanoic acid	M+ACN+H	286.237
	2,4-dimethyl-2E-tetradecenoic acid	M+H	255.232
	Linoelaidic acid	M+H-2H2O, M+H-H2O, M+H	281.246
	3-hydroxypalmitic acid methyl ester	M+H-2H2O, M+K	325.227

9-keto palmitic acid	M+ACN+H	312.254
2Z-octadecenoic acid	M+ACN+H	324.289
Isobutyryl-L-carnitine	M+Na, 2M+3H2O+2H	254.144
Pinolenic acid	M+H	279.232
11-keto palmitic acid	M+H-2H2O	235.205
15-oxo-11Z,13E-eicosadienoic acid	M+H	323.258
(R)-3-Hydroxy-hexadecanoic acid	M+ACN+H	314.268
Stearic acid	M+ACN+H	326.305
9-heptadecylenic acid	M+H-H2O, M+H, 2M+Na	269.247
Alpha-Linolenic acid	M+H	279.232
Palmitoleic acid	M+H	255.231
Gamma-Linolenic acid	M+H	279.231
Pentadecanedioic acid	2M+H	545.404
4E,8E-octadecadienoic acid	M+H-2H2O, M+H-H2O, M+H	263.236
16-methyl-5Z,9Z-octadecadienoic acid	M+H	295.262
Malvalic acid	M+H-2H2O	245.225
5,8,11-Eicosatrienoic acid	M+H	307.263
Linoleic acid	M+H-2H2O, M+H-H2O, M+H	263.236
7,10,13-Eicosatrienoic acid	M+H	307.262
(Z)-9-Heptadecenoic acid	M+H-H2O, M+H	251.236
DL-7-hydroxy stearic acid	M+ACN+H	342.299
10-oxo-nonadecanoic acid	M+H, M+Na, M+ACN+H	354.297
2-methyl nonaoic acid	M+H-2H2O	137.132
13-Docosenoic acid	M+ACN+H	380.352
15Z-octadecenoic acid	M+H-2H2O, M+H-H2O, M+H	265.252
Heptadecanoic acid	M+H-2H2O	235.241
Mycolipanolic acid (C28)	M+ACN+H	482.457
4-methyl-undecanoic acid	2M+K	439.32
5Z,15Z-eicosadienoic acid	M+H-H2O, M+H	309.278
20-hydroxy-eicosanoic acid	M+ACN+H	370.332
omega-hydroxy behenic	M+ACN+H	398.362

	2-hydroxy-heneicosanoic acid	M+Na, M+ACN+H, M+ACN+Na	384.346
	7-methyl caprylic acid	M+H-2H2O	123.116
	18-oxo-nonadecanoic acid	M+H	313.273
**Lipids and derivatives**	ar-Artemisene	M+2Na-H	315.206
	(20S,22E)-3β-Hydroxy-5α-chol-22-en-24-oic Acid	M+ACN+H	416.316
	1alpha,25-dihydroxy-21-norvitamin D3 / 1alpha,25-dihydroxy-21-norcholecalciferol	M+ACN+H	444.346
	2-ethylacryloylcarnitine	M+H-2H2O, M+H, M+Na	266.15
	(11Z,14Z)-eicosadienoylcarnitine	M+H-2H2O	416.353
	(+)-Vulgraon B	M+2Na-H	261.159
	Demethyltorosaflavone C	M+2H, M+Na	198.042
	LysoSM(d18:1)	M+H-2H2O	430.332
	(9Z,12Z,15Z)-3-hydroxyoctadecatrienoylcarnitine	M+H-2H2O	402.299
	(5Z)-(3S)-1alpha,25-dihydroxy-3-deoxy-3-thiavitamin D3 3-oxide / (5Z)-(3S)-1alpha,25-dihydroxy-3-deoxy-3-thiacholecalciferol 3-oxide	M+H	435.293
	dodecanamide	M+H	200.2
	Isopetasoside	M+H	397.222
	(4Z,9a)-9-Angeloyloxy-4,10(14)-oplopadien-3-one	M+K	355.167
	N-(3-oxo-hexanoyl)-homoserine lactone	M+Na	178.086
	(+)-Hirusten-12-oic acid	M+H	281.138
	13-(beta-D-glucosyloxy)docosanoic acid	M+H-2H2O, M+2Na-H	483.369
	N-(2E,4E,10E,12Z-tetradecatetraen-8-yn-oyl) isobutylamine	M+2Na-H	316.165
	4,5-Dihydroniveusin A	M+H-2H2O	361.165
	Deacetylisovaltrate	M+H-2H2O	345.171
	(R)-1-O-[b-D-Glucopyranosyl-(1->6)-b-D-glucopyranoside]-1,3-octanediol	M+H-2H2O	435.223
	LysoPE(15:0/0:0)	M+H-2H2O	404.258
	N-stearoyl tyrosine	M+H-2H2O	412.323
	20S-Hydroxycholest-4-en-3,16-dione	M+K	453.277
	Gibberellin A19	M+H-H2O	345.171
	1,2-dihexanoyl-sn-glycero-3-phosphate	M+H-2H2O, M+H-H2O, M+ACN+H	410.228
	N-oleoyl phenylalanine	M+H-2H2O	394.309
	N-stearoyl phenylalanine	M+H-2H2O	396.326
	6,7-Epoxy-3Z,9Z-tricosadiene	M+ACN+Na	398.34
	Tetranor-PGEM-d6	M+H	333.181
	5,9-tetracosadienoic acid	M+ACN+Na	428.351

19-Nor-14-epi-23-yne-1,25 dihydroxyvitamin D3	M+H-H2O	383.296
2R-hydroxy-oleic acid	M+ACN+H	340.284
Arachidonoyl-EA(d8)	M+Na	378.321
PGE1-EA	M+H	398.29
LMFA13010020	M+H-2H2O	539.433
Capsidiol	M+H	237.184
3-Hydroxysintaxanthin	M+H-2H2O	411.304
N-heptanoyl-homoserine lactone	M+H-2H2O	178.122
N-(2-fluro-ethyl) 2-methyl-arachidonoyl amine	M+K	402.257
Taurochenodesoxycholic acid	M+H-2H2O	464.284
26,27-dinor-cholest-5-en-23-yn-3β-ol	M+ACN+H	396.326
2,6-Dimethyl-7-octene-1,6-diol 8-O-glucoside	M+ACN+H	376.234
Annoglabasin C	M+Na	429.223
8-Oxohexadecanoic acid	M+H-2H2O	235.205
Melleolide G	M+ACN+H	474.247
6,7-Dimethoxy-7-epirosmanol	M+ACN+H	432.238
Tetradecanoylcarnitine	M+2Na-H	416.274
C16 Sphinganine	M+H	274.274
5,8,12-TriHOME(9)	M+H-2H2O	295.225
Xestoaminol C	M+H	230.247
Lubiminol	M+H-H2O, M+Na	254.211
N,N,N-trimethyl-sphingosine	M+ACN+Na	406.352
1-Octylglycerol	2M+ACN+H	450.379
terpentecin	M+H-H2O	347.185
Prosopinine	M+H	288.252
24-keto-25dehydrocholestanol	M+ACN+H	442.367
12S-HpEPE	M+H-2H2O	299.2
Tetranor 12-HETE	M+H	267.194
MG(20:5(5Z,8Z,11Z,14Z,17Z)/0:0/0:0)	M+H	377.269
3-ketosphingosine	M+H-H2O	280.263
Cincassiol B	M+H, M+Na	423.198
xi-7-Hydroxyhexadecanedioic acid	M+H-2H2O	267.194
Annosquamosin A	M+H-2H2O	327.231
Etretinate	M+H-H2O, M+H	355.227
5,8-tetradecadienal	M+H-H2O, M+H, M+ACN+H	209.189
12S,13R-EpOME	M+H-H2O, M+H	279.231
(2E)-Dodecenoyl-CoA	M+ACN+H	985.268
1a,1b-dihomo-PGD2	M+H-H2O, M+ACN+H	422.261

Gibberellin A6	M+2Na-H	391.112
Vulgarone A	M+H	219.173
(Z)-13-Oxo-9-octadecenoic acid	M+H-H2O, M+H	279.231
12S-HHTrE	M+H	281.212
Penaresidin A	M+H	330.3
2,5-dimethyl-2E-tridecenoic acid	M+H-H2O, M+H	223.206
3,16-dihydroxypalmitic acid	M+H-2H2O	253.215
Lauroyl-EA	M+H-H2O, M+H, M+Na	244.226
1alpha,25-dihydroxy-24-nor-22-oxavitamin D3 / 1alpha,25-dihydroxy-24-nor-22-oxacholecalciferol	M+H	405.3
(9S,10E,12Z,15Z)-9-Hydroxy-10,12,15-octadecatrienoic acid	M+H-H2O	277.216
12R-HOME(10E)	M+H-2H2O, M+H-H2O, M+H	299.257
Sphinganine	M+H-H2O	284.294
Enigmol	M+H	302.305
Phytocassane D	M+H	317.211
(Â±)-Hydroxycitronellol	2M+ACN+H	390.358
Phytosphingosine	M+H	318.3
(8Z,d18:1) sphingosine	M+H	300.289
19-HOME(9Z)	M+H-2H2O, M+H-H2O, M+H	281.247
2,4-Undecadiene-8,10-diynoic acid isobutylamide	2M+H	459.302
8-hydroxy-11Z-octadecen-9-ynoic acid	M+H-H2O, M+H	295.226
PG(14:1(9Z)/0:0)	M+2H	228.123
9Z,12Z,15Z-Octadecatrienal	M+H-H2O	245.226
12-epi-12-D2t-IsoP	M+H-2H2O	317.212
10E,12E,14E-Hexadecatrienal	M+H	235.205
3-Methylpentadecan-2-one	M+ACN+H	282.279
Ginsenoyne K	M+H	277.18
3-Dehydrosphinganine	M+H	300.289
3alpha,7alpha,12alpha,25-Tetrahydroxy-5beta-cholestane-24-one	M+H-2H2O	415.32
(9R,13R)-10-oxo-11-phytoenoic acid	M+H-H2O, M+H	277.216
(E)-3-decen-1-ol	2M+ACN+H	354.336
(E)-2-Octen-1-ol	2M+Na	279.23
Prostaglandin F1a	M+H-2H2O	321.241

1,2-dimethylcyclopentane	2M+Na	219.208
(2'E,4'Z,8E)-Colneleic acid	M+H-H2O, M+Na	277.216
LysoSM(d18:0)	M+H, M+Na	489.342
(4E,8E,10E-d18:3)sphingosine	M+H	296.258
LMFA11000660	2M+Na	219.209
N,N-dimethyl-Safingol	M+H-H2O	312.325
(9S,13S)-10-oxo-11-phytoenoic acid	M+H-H2O, M+H	295.226
1,5Z-Octadien-3-ol	2M+H	253.216
14S-Methyl-1-octadecene	M+ACN+H	308.331
Glycerol tributanoate	M+H-H2O, M+H	303.18
Pentadecanoyl-EA	M+H-H2O, M+H	286.274
Geranyl hexanoate	M+H-H2O, M+H	235.205
(9S,13S)-10,11-dihydro-12-oxo-15-phytoenoic acid	M+H-H2O, M+H, 2M+ACN+H	277.215
Methyl (3x,10R)-dihydroxy-11-dodecene-6,8-diynoate 10-glucoside	M+Na	421.145
5β-Cholane-3α,7α,12α-triol	M+Na	401.303
Stearaldehyde	M+ACN+H	310.311
18-Nor-4(19),8,11,13-abietatetraene	M+H	255.21
punaglandin 7	M+H	439.189
Epinepetalactone	M+H-2H2O	131.085
PS(17:1(9Z)/22:0)	M+Na	854.585
PE(19:0cycv8c/17:0cycw7c)	M+Na	766.533
PG(19:iso/12:0)	M+ACN+H	750.531
1-hexadecyl-2-palmitoyl-glycero-3-phosphoethanolamine	M+2Na-H	722.506
Dehydroandrosterone	2M+ACN+H	618.454
PGF2alpha isopropyl ester	M+Na	419.277
DG(10:0/10:0/0:0)	M+ACN+H	442.351
Kurilensoside F	M+H-2H2O	595.383
DG(10:0/10:0(3-OH)/0:0)	M+ACN+H	458.347
(25S)-5α-cholestan-3β,4β,6α,8β,15α,16β,26-heptol	M+Na	507.33
12-Methyltridecanal	M+ACN+H	254.247
Palmitaldehyde	M+Na	263.235
Catalpol	M+ACN+Na	426.137
Penazetidine A	M+ACN+Na	433.378
26,27-diethyl-1alpha,25-dihydroxy-22-thia-20-epivitamin D3 / 26,27-diethyl-1alpha,25-dihydroxy-22-thia-20-epicholecalciferol	M+H-H2O	473.345

Tetradecanal	M+ACN+H	254.247
5'-O-Methylmelledonal	M+K, M+ACN+H	485.158
meso-Pristane	M+ACN+H	310.347
1alpha,25-dihydroxy-26,27-dimethyl-24a-homo-22-thia-20-epivitamin D3 / 1alpha,25-dihydroxy-26,27-dimethyl-24a-homo-22-thia-20-epicholecalciferol	2M+3H2O+2H	490.344
(22E, 24x)-Ergosta-4,6,8,22-tetraen-3-one	2M+ACN+Na	848.629
Tetranor-PGF1alpha	M+ACN+Na	364.21
LysoPE(18:0/0:0)	M+H	482.323
Serratamic acid	2M+3H2O+2H	289.184
PA(13:0/12:0)	M+2Na	298.171
N-(tetradecanoyl)-deoxysphing-4-enine-1-sulfonate	M+H-H2O	556.442
1,3-dipentadecanoyl-2-hydroxy-glycerol (d5)	M+K	584.47
1alpha-fluoro-25-hydroxy-16,17,23,23,24,24-hexadehydrovitamin D3 / 1alpha-fluoro-25-hydroxy-16,17,23,23,24,24-hexadehydrocholecalciferol	M+H, M+Na	413.284
(25R)-26,26,26-trifluoro-1alpha,25-dihydroxyvitamin D3 / (25R)-26,26,26-trifluoro-1alpha,25-dihydroxycholecalciferol	M+K	509.265
16,17-Dihydro-16a,17-dihydroxygibberellin A7 17-glucoside	M+K	565.17
Corchoionol C 9-glucoside	M+2Na-H	431.166
Vetiverol	M+H-H2O, M+H	221.189
Calcidiol	M+H-H2O, 2M+K	383.33
Dihydrophytol	M+ACN+H	340.357
1alpha,25-dihydroxy-24a-homo-22-thia-20-epivitamin D3 / 1alpha,25-dihydroxy-24a-homo-22-thia-20-epicholecalciferol	M+H-2H2O	413.287
13S-hydroxyoctadecadienoic acid	M+H-H2O	279.231
Panaxatriol	M+ACN+Na	540.402
2-amino-14,16-dimethyloctadecan-3-ol	M+H	314.341
9S,11R,15S-trihydroxy-2,3-dinor-13E-prostaenoic acid-cyclo[8S,12R]	M+K	367.188
cyasterone	M+H-2H2O	485.289
DG(10:0(3-OH)/12:0(3-OH)/0:0)	M+ACN+H	502.372
PA(18:4(6Z,9Z,12Z,15Z)/0:0)	M+H-H2O	413.208
Campestanol	M+H-2H2O	367.371
Miltirone	M+H	283.169
Isorenieratene/ (Leprotene)	M+ACN+Na	592.389
1-tridecanoyl-sn-glycero-3-phosphate	M+Na	391.185
2-Octaprenyl-3-methyl-6-methoxy-1,4-benzoquinol	M+K	603.417

Glycerol 1-octadecanoate	M+K	397.272
Cholic acid	M+ACN+H	450.321
7-Dehydrodesmosterol	M+H-H2O	365.32
Perilloside B	M+H-H2O	311.15
Spirilloxanthin	M+K	635.424
2-Decaprenyl-6-methoxyphenol	M+K	843.642
TG(17:0/17:2(9Z,12Z)/18:4(6Z,9Z,12Z,15Z))[iso6]	M+Na	873.697
5a-Androst-3-en-17-one	2M+K	583.392
1-(13Z,16Z-docosadienoyl)-glycero-3-phospho-(1'-sn-glycerol)	M+H	565.352
Muricholic acid	M+ACN+H	450.321
Menthol	M+Na	179.141
[6]-Gingerdiol 3,5-diacetate	M+ACN+H	422.253
xi-p-Menth-3-ene	M+Na	161.13
13,17,21-trimethyl-5Z,9Z,19Z-docosatrienoic acid	M+Na	399.324
MG(16:0/0:0/0:0)	M+H-2H2O, M+ACN+Na	295.262
5a-Cholesta-8,24-dien-3-one	M+H-H2O	365.32
Cembrene	M+H	273.257
CDP-DG(14:0/14:0)	M+ACN+Na	961.467
1alpha-hydroxy-18-(5-hydroxy-5-methyl-2-hexynyloxy)-23,24,25,26,27-pentanorvitamin D3 / 1alpha-hydroxy-18-(5-hydroxy-5-methyl-2-hexynyloxy)-23,24,25,26,27-pentanorcholecalciferol	M+H-H2O, M+Na, M+ACN+Na	439.32
13'-Carboxy-alpha-tocopherol	M+ACN+H	502.389
Hydroxysintaxanthin 5,6-epoxide	M+H-2H2O	427.3
Butyl butyryllactate	2M+Na	455.262
2,3-Dinor-6-keto-prostaglandin F1 a	M+Na	365.193
1alpha,25-dihydroxy-11alpha-[(1R)-oxiranyl]vitamin D3 / 1alpha,25-dihydroxy-11alpha-[(1R)-oxiranyl]cholecalciferol	M+ACN+Na	522.357
Cholesterol-5beta-hydroperoxide	M+H-H2O	401.34
1-eicosyl-glycero-3-phospho-(1'-myo-inositol)	M+H-2H2O	579.368
26-O-[β-D-glucopyranosyl]-25R-furostan-3β,22α,26-triol	M+H	597.402
2E-Phytenal	M+ACN+H	336.325
DG(18:0/15:0/0:0)	M+H-H2O	565.518
Corchorusoside B	M+H-2H2O	647.345
LBPA(16:0/18:1(9Z))	M+H-2H2O	713.508
TG(22:4(7Z,10Z,13Z,16Z)/20:1(11Z)/18:4(6Z,9Z,12Z,15Z))	M+H	957.792
1-Undecanol	2M+ACN+H	386.4
1-tetradecanoyl-glycero-3-phospho-(1'-sn-glycerol)	M+H-2H2O	421.235
NeuAcα2-3Galβ-Cer(d18:1/24:1(15Z))	M+H+Na	562.385

Archaetidylglycerol-(glycosyl)-myo-inositol	M+H+Na	540.373
11alpha-(4-dimethylaminophenyl)-1alpha,25-dihydroxyvitamin D3 / 11alpha-(4-dimethylaminophenyl)-1alpha,25-dihydroxycholecalciferol	2M+3H2O+2H	549.412
MG(0:0/22:2(13Z,16Z)/0:0)	M+H	411.346
1-hexadecanoyl-sn-glycero-3-phospho-(1'-sn-glycerol)	M+2H	243.148
(25S)-5α-cholestan-3β,6α,7β,8β,15α,16β,26-heptol	M+H, 2M+H	969.671
Trihydroxycoprostanoic acid	M+ACN+H	506.383
Ganoderic acid Mf	M+H-H2O	495.347
Cholesta-4,6-dien-3-one	M+H-H2O	365.32
2,6,14-trimethyl-10-methylene-9-(3-methylpent-4-enyl)-pentadec-6E-ene	M+2Na-H	391.331
Glycerol triundecanoate	M+H	597.509
4b-Hydroxycholesterol	M+H-2H2O	367.336
Furopirostane skeleton	M+H-2H2O	365.321
Cer(d18:0/20:0)	M+H	596.598
DG(22:6(4Z,7Z,10Z,13Z,16Z,19Z)/22:6(4Z,7Z,10Z,13Z,16Z,19Z)/0:0)	M+H-2H2O	677.496
Elaterinide	M+ACN+H	760.393
Pisumoside A	M+2Na-H	899.347
PG(14:0/16:0)	2M+3H2O+2H	708.489
CL(18:0/18:0/16:1(9Z)/16:1(9Z))	M+2H	703.51
PS(12:0/22:2(13Z,16Z))	M+H	760.515
24:2(5Z,9Z)(11Me,15Me,19Me,23Me)	M+Na	443.387
Minabeolide-5	M+H-H2O	437.267
5-Androstenetriol	M+H-2H2O	271.205
1-(9Z-tetradecenoyl)-2-(9Z-pentadecenoyl)-glycero-3-phosphoserine	M+2Na-H	734.4
Glycerol 2-(9Z,12Z-octadecadienoate) 1-hexadecanoate 3-O-[alpha-D-galactopyranosyl-(1->6)-beta-D-galactopyranoside]	M+ACN+H	958.646
MG(0:0/16:0/0:0)	M+H-H2O	313.273
2'-O-(α-D-Manp)-(1-(9Z,12Z-octadecadienoyl)-sn-glycero-3-phospho-1'-myo-inositol)	M+ACN+H	800.385
15-Hentriacontanol	M+2Na-H	497.467
(3beta,5alpha,6beta,9alpha,22E,24R)-23-Methylergosta-7,22-diene-3,5,6,9-tetrol	M+H	461.363
1,2-di-(11Z-docosenoyl)-sn-glycero-3-phospho-(1'-sn-glycerol)	M+H, M+2H, M+Na, M+H+Na	909.65
N-(9Z-octadecenoyl)-tetradecasphing-4-enine	M+H	508.471
Thromboxane B2	M+ACN+Na, 2M+3H2O+2H	434.258
24S,25-dihydroxycholesterol	M+H-H2O	401.341

	Glycerol 1,3-didodecanoate 2-decanoate	M+H	611.525
	Ubiquinone-1	M+H-2H2O	215.105
	DG(10:0/15:0/0:0)	M+H-H2O, M+H, M+Na	453.393
	Cer(d18:0/12:0)	M+H	484.471
	9-Hexadecenoylcarnitine	M+ACN+H	439.353
	phorbol 13-acetate 12-myristate	M+H-2H2O	581.386
**Organic acids and derivatives**	L-2,3-Dihydrodipicolinate	2M+3H2O+2H, 2M+ACN+H	183.048
	4'-phosphopantetheine	M+2Na	200.04
	2-Oxosuccinamate	M+Na	154.011
	Isobutyl isobutyrate	M+H-2H2O, M+ACN+Na	208.132
	Acetaminophen cystein	M+H-H2O	237.069
	Neoherculin	M+ACN+Na	311.21
	(R)-3-Hydroxy-5-phenylpentanoic acid	M+H-2H2O	159.08
	3-Sulfinoalanine	M+ACN+H	194.035
	Anofinic acid	M+ACN+H	246.112
	Ergothioneine	M+ACN+Na	293.105
	Hydrogen carbonate	2M+ACN+H	164.019
	Î²-D-glucose 6-phosphate	M+H-2H2O	221.02
	N-Lauroylglycine	M+Na	280.188
	Palmitoylglycine	M+H	314.269
	Artemisyl propionate	M+H	211.168
	S-(3-Methyl-2-butenyl) 2-methylpropanethioate	M+ACN+H	214.125
	Dihydroceramide	M+H	330.3
	N1,N8-Diacetylspermidine	M+ACN+H	272.221
	Tricosanoylglycine	M+2Na-H	456.343
	N2-(2-Carboxymethyl-2-hydroxysuccinoyl)arginine	M+ACN+H	390.162
	Domoic acid	M+ACN+H	353.172
	Argininic acid	2M+ACN+Na	414.207
	2,4-Hexadienyl acetate	M+H-2H2O	105.069
	Dopaxanthin quinone	M+ACN+Na	452.105
	L-glycyl-L-hydroxyproline	2M+ACN+H	418.193
	Acetyl tributyl citrate	M+H	403.232
	Neuromedin N	M+H-2H2O	582.364
	Fumonisin A2	M+H	748.411
	Mytilin A	M+2Na-H	377.093
	Na-Hexanoyl-Nb-inosityltryptophan	M+2Na-H	509.186
**Organic compounds**	Methyl bisnorbiotinyl ketone	M+H-2H2O	179.063
	3-Amino-1,4-dimethyl-5H-pyrido[4,3-b]indole	M+H	212.117
	Lactarofulvene	M+2Na-H	241.096
	Gamma-glutamyl-L-putrescine	M+Na	240.132

1,3-Octadiene	M+2Na-H	155.08
1-Dodecene	M+2Na-H	213.158
N-trans-p-Coumaroyloctopamine	M+Na, M+K, M+ACN+Na	338.08
Oxalureate	2M+ACN+Na	328.051
Marshdine	M+K	338.042
Hexahydro-6,7-dihydroxy-5-(hydroxymethyl)-3-(2-hydroxyphenyl)-2H-pyrano[2,3-d]oxazol-2-one	M+2Na-H	342.056
Gomphidic acid	M+H-H2O	355.046
N-Isobutyl-2,4,8-decatrienamide	M+2Na-H	266.149
(+)-1-Methylpropyl 3-(methylthio)-2-propenyl disulfide	M+ACN+Na	272.057
Tetrahydrofurfuryl cinnamate	M+2Na	139.044
9-Azabicyclo[3.3.1]nonan-3-one	M+ACN+Na	203.116
Albafuran B	M+2Na	212.081
cyclic Melatonin	M+H, M+Na	231.112
Neotame	M+H+Na	201.106
Isoeugenol benzyl ether	M+ACN+Na	318.146
Medroxyprogesterone	M+K	383.198
Aflatoxin G2a	M+Na	369.058
(+/-)-N,N-Dimethyl menthyl succinamide	M+2Na-H	213.159
Harmol	M+ACN+H	240.113
8-O-Methyloblongine	M+ACN+Na	392.207
S-Adenosylmethionine	M+H-2H2O	364.133
Kanzonol F	M+H-2H2O	385.179
Hydroxypropyl cellulose	M+H	266.081
Neurine	M+ACN+Na	167.116
Vildagliptin	M+H-H2O, M+ACN+H	286.19
N2-(gamma-Glutamyl)-4-carboxyphenylhydrazine	M+ACN+H	323.135
4-Ethyl-2-methoxyphenol	2M+Na	327.156
m-Cymene	M+2Na-H	207.112
Nigellicine	M+H-H2O	229.097
Urolithin A-3-O-glucuronide	M+H-2H2O	369.061
Carbinoxamine	2M+K	619.201
7-Hydroxy-2-methyl-4-oxo-4H-1-benzopyran-5-carboxylic acid 7-glucoside	M+2H	192.052
Benzoyl glucuronide (Benzoic acid)	2M+3H2O+2H, 2M+ACN+Na	312.086
Chalconaringenin 2'-rhamnosyl-(1->4)-glucoside	M+2Na	313.078
(2S,4R)-4-(9H-Pyrido[3,4-b]indol-1-yl)-1,2,4-butanetriol	M+ACN+H	314.15
Pelargonidin 3-O-[2-O-(β-D-xylopyranosyl)-6-O-(methyl-malonyl)-bata-D-galactopyranoside]	M+2H	333.592
Petunidin 3-rutinoside	M+2Na-H	670.149
Benzyl methyl disulfide	M+H, M+Na	171.03

3,4,17-trihydroxy-9,10-seco-androsta-1,3,5(10)-triene-9-one	M+H	319.19
Isopropyl apiosylglucoside	M+H-H2O	337.151
2-Propyl-2,4-pentadienoic acid	M+H-2H2O	105.07
(4-Hydroxy-3-methoxyphenyl)ethanol	M+H-2H2O	133.064
2,3,6,7-Tetrahydrocyclopent[b]azepin-8(1H)-one	M+H-H2O, M+H	150.09
(4-Hydroxybenzoyl)choline	M+K	263.092
2-Heptylbenzothiazole	M+K	272.087
9-(beta-D-Ribofuranosyl)zeatin	M+H	352.16
Methcathinone	2M+ACN+H	368.232
3-Ethyl-1,2-cyclopentanedione	M+H-2H2O	91.0545
(6E,8E)-4,6,8-Megastigmatriene	M+2Na-H	221.127
Subaphylline	M+H-H2O	247.144
alpha-Hydrojuglone 4-O-b-D-glucoside	M+2Na-H	383.07
3-Isopropyl-2-methoxy-5-methylpyrazine	M+H, M+Na, M+ACN+Na	230.128
1,1-Dimethoxyoctane	M+ACN+H	216.195
xi-1-Butoxy-1-methoxyethane	2M+ACN+H	306.263
Koeniginequinone A	M+ACN+H	283.108
(S)-5'-Deoxy-5'-(methylsulfinyl)adenosine	2M+3H2O+2H	327.095
alpha-Hydroxy-1-methyl-1H-indole-3-propanoic acid	M+Na	242.08
4-Hydroxy-5-(3',5'-dihydroxyphenyl)-valeric acid-O-glucuronide	M+H	403.122
2-Phenylethyl 2-aminobenzoate	M+H	242.117
Torasemide	M+Na, M+ACN+H	390.142
Mangostinone	M+ACN+H	422.198
3-Acetylpyridine	M+H	122.06
4,5-Dimethyl-2-heptyloxazole	M+ACN+Na	259.179
Zanthosimuline	M+Na	332.162
1-Deoxy-L-glycero-tetrulose 4-phosphate	M+Na	207.003
1-Phenyl-1,3-nonadecanedione	M+ACN+H	414.336
Biphenyl	M+K	193.042
3'-Deaminofusarochromanone	M+H-2H2O	242.117
2-[(2-Furanylmethyl)thio]-6-methylpyrazine	M+H	207.058
1,18-Diamino-5,9,14-triazaoctadecane	M+K	326.268
Ketophenylbutazone	M+ACN+H	364.166
Methoxybrassitin	M+H-2H2O	215.063
4-Hydroxybenzeneacetonitrile	2M+ACN+Na	330.122
4-(Î³-glutamylamino)butanal	2M+3H2O+2H	229.116
Zileuton	M+H	237.069
gamma-Glutamyl-gamma-butyraldehyde	M+Na	239.1
4-Methyl-2-propyloxazole	2M+H	251.174

Amsacrine	M+H, M+K	389.161
9H-Carbazole-3-carboxaldehyde	2M+Na	413.126
Sofalcone	M+H-2H2O, M+H+Na	237.101
N-Acetylserotonin	M+H-H2O	201.102
3-Hydroxy-carbofuran	M+H-2H2O	202.086
Heptanoylcholine	M+2Na-H	261.168
Kamahine C	M+2Na-H	313.101
N6-cis-p-Coumaroylserotonin	M+ACN+H	364.166
Propyl cinnamate	M+ACN+H, 2M+Na	403.177
1,2,5,6-Tetrahydro-4H-pyrrolo[3,2,1-ij]quinolin-4-one	M+ACN+H	215.117
1,17-Diamino-4,9,13-triazaheptadecane	M+K	312.253
4-Hydroxybenzyl isothiocyanate	M+ACN+H	207.058
2-(Methylthio)-3H-phenoxazin-3-one	M+ACN+H	285.069
Terodiline hydrochloride	M+H-H2O	299.18
2-Phenyl-4-pentenal	M+ACN+H	202.122
[10]-Dehydrogingerdione	M+H-2H2O	311.199
14,19-Dihydroaspidospermatine	M+H	341.222
10,12-Heptacosanedione	M+K	447.36
3-Phenoxybenzoic acid	M+H	215.07
3-Methylcyclohexanol	2M+ACN+H	270.242
Erucoylacetone	M+ACN+Na	442.366
Acetal R	M+ACN+H	250.18
alpha-Butyl-omega-hydroxypoly(oxyethylene) poly(oxypropylene)	M+Na	271.187
Pongamoside A	M+K	479.075
3',4',5'-Trimethoxycinnamyl alcohol acetate	M+H, M+2Na-H	267.122
3,5-Di-O-methyl-8-prenylafzelechin-4beta-ol	M+K	425.137
3-Methyl-5-pentyl-1,2,4-trithiolane	2M+3H2O+2H	222.053
Ethyl maltol	2M+H	281.102
4,5-Di-O-methyl-8-prenylafzelechin-4beta-ol	M+H-H2O, M+H, M+Na	387.18
(+)-Setoclavine	M+H-H2O	237.138
N-[(4-Hydroxy-3-methoxyphenyl)methyl]octanamide	M+H-H2O	262.18
Cyclobrassinin	M+H	235.035
1,1-Diethoxypentane	2M+ACN+H	362.326
DG(14:0(3-OH)/17:0cycw7c/0:0)	M+2Na-H	613.441
Batatasin III	M+ACN+H	286.143
2-Pentyl-3-phenyl-2-propenal	2M+K	443.234
N,N,O-Tridesmethylvenlafaxine	M+H-H2O	218.153
5-Methoxy-7,8-diprenylflavone	M+Na	411.194
12,14-Nonacosanedione	M+K	475.392

Purpuritenin B	2M+ACN+H	626.256
Cotinine methonium ion	M+Na, M+ACN+H	233.153
Nitecapone	M+H	266.067
2,5-Dibenzyl-3-hydroxy-6-methoxypyrazine	M+H-2H2O	271.124
8,8-Diethoxy-2,6-dimethyl-2-octanol	M+ACN+H	288.253
Lotaustralin	M+H-H2O	244.118
Moringyne	M+ACN+Na	376.137
Chondroitin D-glucuronate	2M+ACN+Na	882.333
D8'-Merulinic acid C	M+H-H2O	357.278
Oxybenzone	M+H	229.085
Norambreinolide	M+H	251.2
2-Pentadecanone	M+ACN+H	268.263
Deoxyeritadenine	M+H-H2O	220.083
N-Desmethylaminopyrine	M+ACN+Na	281.137
(S)-(E)-8-(3,6-Dimethyl-2-heptenyl)-4',5,7-trihydroxyflavanone	M+H	397.201
N5-Formyl-THF	M+2Na-H	518.137
Resolvin E1	M+H-2H2O	315.195
Kushenol P	M+H	457.222
(2S,4S,6S)-2-[2-(4-Hydroxy-3-meyhoxyphenyl)ethyl]tetrahydro-6-(4,5-dihydroxy-3-methoxyphenyl)-2H-pyran-4-ol	M+2Na-H	435.14
Phenylethylamine	2M+H	243.185
Cotinine glucuronide	M+ACN+Na	416.143
Neoacrimarine B	M+H	668.284
Neocretanin	M+K	507.056
Calomelanol B	M+H, M+ACN+H	405.135
Acetaldehyde hexyl isoamyl acetal	M+ACN+H	258.242
2-Heptadecanone	M+ACN+H	296.257
N1-trans-Feruloylagmatine	M+ACN+Na	370.185
(R)-Kanzonol Y	M+H+Na	217.103
Lipoyllysine	M+H-2H2O	299.125
Memantine	M+ACN+Na	243.184
Ethylene brassylate	M+H, M+2Na-H	315.18
Dehydrophytosphingosine	M+H-H2O	298.273
heptadecanal	M+ACN+H	296.295
1,1-Dimethoxynonane	M+ACN+H	418.389
Palmitoleoyl Ethanolamide	M+H-H2O, M+H	280.263
Pteroside P	M+2Na	221.079

D6-Ambrettolide	M+H-H2O, M+H	253.215
Pteroside D	M+ACN+Na	474.21
Cycloartomunoxanthone	M+Na, M+ACN+H	490.159
1-Nonadecene	M+ACN+H	308.331
5-Methoxy-7,8-methylenedioxyflavone	M+2H	149.041
Deoxythymidine diphosphate-l-rhamnose	M+H	549.087
Harmanine	2M+Na	419.148
4-Hydroxydiphenylamine	2M+H	371.175
Longicaudatin	M+H	447.143
1-Octene	2M+Na	247.24
9Z-Nonadecene	M+ACN+H	308.33
3Z,6Z,9Z-Heneicosatriene	M+ACN+H	332.331
decanamide	2M+K	381.289
9'-Carboxy-gamma-chromanol	2M+ACN+H	794.557
Nonoxynol-9	M+Na	639.409
10,12-Tritriacontanedione	M+K	531.452
1-Methoxy-3-(4-hydroxyphenyl)-2E-propenal 4'-glucoside	M+ACN+H	368.171
1,1'-Bis(2-hydroxy-3-methylcarbazole)	M+ACN+H	434.188
Delphinidin 3-(acetylglucoside)	M+2Na-H	552.087
(5alpha,8beta,9beta)-5,9-Epoxy-3,6-megastigmadien-8-ol	M+H-2H2O, 2M+H	417.301
3-Hydroxyphenanthrene	2M+Na	411.136
4-Hydroxyphenanthrene	2M+ACN+Na	452.161
Terbinafine	M+ACN+Na	355.214
10-Methyltridecan-2-one	M+Na	235.203
2S-Hydroxyoctan-3-one	M+H-2H2O	109.101
shikimate 3-phosphate	M+Na	273.985
7-Hydroxy-3',4'-methylenedioxyflavan 7-O-β-D-glucopyranoside	M+2Na-H	477.113
5-Acetylamino-6-formylamino-3-methyluracil	2M+Na	475.128
Glycineamideribotide	M+H-2H2O	251.042
[12]-Gingerol	M+Na	401.266
Kanzonol N	M+ACN+Na	448.173
Benzyl cinnamate	2M+Na	499.187
Nonadecane	M+ACN+H	310.346
Tridecanamide	M+H-H2O	196.205
Linoleamide	2M+H	559.521
1-Undecene	M+2Na	100.075
Dihydrocaffeic acid 3-O-glucuronide	M+ACN+H	400.124
Quercetin 3-(6"-malonyl-glucoside)	M+H-H2O	533.093

Artocommunol CA	M+H, M+ACN+H	433.166
Isoeugenol phenylacetate	2M+Na	587.24
5,9-Hexacosadiene	M+Na	385.379
Nonadecanal	M+ACN+H	324.326
N-methyl arachidonoyl amine	M+ACN+H	359.305
Batatasin IV	2M+H	489.227
2',4',6',3-Tetrahydroxy-3'-geranyl-6",6"-dimethylpyrano[2",3":4,5]dihydrochalcone	M+ACN+H	534.285
2,6-Di-tert-butyl-4-ethylphenol	M+H, 2M+ACN+H	510.432
3-Methylheptane	2M+ACN+H	270.315
5,8-Epoxydaucane	M+H-H2O, M+H	223.204
2-Dodecylbenzenesulfonic acid	M+H	327.198
Margaroyl-EA	M+H	314.306
Octadecylamine	M+H	270.315
N-Acetyldehydroanonaine	M+ACN+Na	369.12
Lacto-N-biose I	M+H	384.15
Citalopram N-oxide	M+H	341.164
2,3-Dehydrosalvipisone	M+H	311.164
Cis-zeatin	2M+ACN+Na	502.242
5-Dodecyldihydro-2(3H)-furanone	M+H-H2O, M+H	237.22
Hexanal hexyl isoamyl acetal	M+ACN+H	314.305
(+/-)N-(1-methyl-2-hydroxy-2-phenyl-ethyl) arachidonyl amine	2M+ACN+H	916.694
7Z,11E-Hexadecadienal	M+H-H2O, M+H	237.221
Kessyl glycol	M+H-2H2O, 2M+ACN+H	219.173
2-(Arabinosylamino)-3-(glucosylamino)propanenitrile	M+H	380.166
9S,10R-Epoxy-3Z,6Z-eicosadiene	M+K, M+ACN+Na	331.239
2,4,12-Octadecatrienoic acid piperidide	M+ACN+H	387.336
Pipereicosalidine	M+ACN+Na	437.352
12,13S-EOT	M+ACN+H	334.237
(Cyclohexylmethyl)pyrazine	M+Na	199.121
N-propyl arachidonoyl amine	M+ACN+H	387.336
Malvidin 3-(6-acetylglucoside)	M+2Na-H	580.118
N,N-Dimethylsphingosine	M+H	328.321
1-Octacosene	M+2Na-H	437.409
Cellulose, microcrystalline	M+ACN+H	412.181

5-(12-Heneicosenyl)-1,3-benzenediol	M+H-2H2O, M+H-H2O, M+H	385.346
Amylose	M+ACN+H	412.181
1-Pentadecene	M+Na	233.225
N-palmitoyl serine	M+H-H2O	326.269
tetrahydropteroyl mono-L-glutamate	M+ACN+H	485.189
b-D-fructosyl-a-D-(6-O-(E))-feruloylglucoside	M+Na	495.146
5-Nonadecyl-1,3-benzenediol	M+Na	399.324
1,1'-[1,13-Tridecanediylbis(oxy)]bisbenzene	M+H-H2O, M+H	369.296
N-palmitoyl leucine	M+H	370.331
L-Olivosyl-oleandolide	M+H-H2O	499.292
Cinnamyl phenylacetate	2M+Na	527.219
Dipiperamide E	M+H-H2O, M+H, M+2Na-H, 2M+Na	571.245
Sialyl-Lewis X	M+Na	843.284
Tamsulosin	2M+K	855.311
alpha-Ionene	2M+K	387.243
Adrenoyl ethanolamide	M+ACN+Na	439.329
1,5,5,8-Tetramethyl-12-thiabicyclo[9.1.0]dodeca-3,7-diene	2M+ACN+H	514.354
2,6,6-Trimethyl-1-cyclohexen-1-acetaldehyde	2M+H	333.277
11-dehydro-TXB2-d4	M+2Na-H	417.218
12-oxo-PDA	2M+ACN+H	626.443
4-Prenylresveratrol	2M+Na	615.272
Pibutidine	M+H-H2O	339.182
Megastigmatrienone	2M+K	419.234
Oxacyclotetradecan-2-one	M+H	213.184
N-oleoyl leucine	M+H	396.346
Myristoleyl myristoleate	M+Na	443.387
Docosahexaenoyl Ethanolamide	2M+ACN+H	784.602
PE(15:0/12:0(3-OH))	M+2Na-H	682.4
undecyl butyrate	M+H	243.231
3-Nonyl-1H-pyrazole	2M+Na	411.346
DG(10:0/12:0(3-OH)/0:0)	M+K	483.308
Isoricinoleic Acid	2M+H	597.509
18Z,21Z-Heptacosadien-10-one	M+Na	413.376
Alpha-Linoleoylcholine	M+2Na-H	411.308
formyl 7E-hexadecenoate	M+H	269.247
Biperiden	2M+ACN+Na	686.464
Secoisotetrandrine	M+H, M+Na, M+ACN+Na	653.284

	Riboflavine 2',3',4',5'-tetrabutanoate	M+ACN+H	698.341
	Pandamarilactone 31	2M+ACN+Na	726.372
	Monensin A	M+H, M+2H	336.223
	Serotonin	2M+ACN+H	394.225
	Eicosanoyl-EA	M+H, M+Na	356.352
	(Z,Z,Z)-11,14,17-Eicosatrienyl 4-methylvalerate	M+Na	413.339
	3-Methyltricosane	M+2Na-H	383.362
	N-Heptane	2M+ACN+Na	264.267
	4Î±-carboxy-5Î±-cholesta-8,24-dien-3Î²-ol	M+H-H2O	410.317
	4,6-Pentacosanedione	M+Na	403.353
	2-Methyldocosane	M+ACN+H	366.408
	2-Hydroxy-24-keto-octacosanolide	M+H, M+ACN+H	453.393
	9,10-Hexacosadiene	M+2Na-H	407.363
	N-[2-(1H-Indol-3-yl)ethyl]docosanamide	M+Na	505.415
	2-(9R-(15Z-docosenoyloxy)-3-methyl-2Z-decenoyloxy)-ethanesulfonic acid	M+H-2H2O, 2M+3H2O+2H	642.453
	2-Hydroxy-2-methylheptan-4-one	M+H-2H2O	109.101
	1,2,3,4-Tetrahydro-1-phenyl-4-(1-phenylethyl)naphthalene	M+Na, 2M+ACN+H	666.37
	3-Amino-1-methyl-5H-pyrido[4,3-b]indole	2M+K	433.154
	PE(12:0(3-OH)/16:1(9Z))	M+2Na-H	694.401
	2-Heptenal	M+H-H2O	95.0856
	cinnamoyl-CoA	M+K	936.12
	di-trans,octa-cis-undecaprenyl phosphate	M+ACN+H	888.697
	Hovenolactone	M+H-H2O	471.347
	Homodihydrocapsaicin	M+ACN+Na	385.246
	2,3-dimethyloctane	2M+ACN+H	326.378
**Pesticide**	Methomyl	2M+3H2O+2H	176.058
	Cyromazine	M+H-H2O, M+ACN+Na	230.102
